# Argus: A Sparse-Label Machine-Learning Workflow for Passive DAS Seismic Catalogue Expansion in CO_2_ Storage Monitoring—Application to the CO2CRC Otway Stage 4 Dataset

**DOI:** 10.3390/s26165084

**Published:** 2026-08-11

**Authors:** Ilgiz Almukhametov, Olivia Collet, Boris Gurevich, Roman Isaenkov, Pavel Shashkin, Konstantin Tertyshnikov, Mikhail Vorobev, Nepomuk Boitz, Roman Pevzner

**Affiliations:** 1 Centre for Exploration Geophysics, School of Earth and Planetary Sciences, Curtin University, GPO Box U1987, Perth, WA 6845, Australia; olivia.collet@curtin.edu.au (O.C.); b.gurevich@curtin.edu.au (B.G.); roman.isaenkov@curtin.edu.au (R.I.); mikhail.vorobev@postgrad.curtin.edu.au (M.V.); 2 Division of Geophysics, Freie Universität Berlin, Malteserstr. 74-100, 12249 Berlin, Germany; nboitz@zedat.fu-berlin.de

**Keywords:** distributed acoustic sensing (DAS), passive seismic monitoring, CO_2_ storage, sparse-label machine learning, feature-space retrieval, positive–unlabelled learning, similarity search, human-in-the-loop, induced seismicity

## Abstract

Passive distributed acoustic sensing (DAS) is an attractive tool for monitoring geological CO_2_ storage, but its dense, continuous recordings create a data-volume problem: a multi-month, multi-well archive yields enormous numbers of detector triggers, of which confirmed seismic events form a vanishingly small fraction, and conventional supervised classification is ill-posed when labels remain scarce and the negative class undefined because the non-event population is open-ended and spans noise families that vary over time and between wells. We present Argus, a sparse-label machine-learning workflow that converts continuous DAS recordings into a reproducible, auditable catalogue of event candidates. A deterministic front end reduces the archive to comparable trigger objects, each described by a 67-feature interpretable representation of its two-dimensional time–channel character (e.g., duration and channel span, detector-mask morphology, apparent moveout, inter-channel waveform coherence, and spectral shape); a retrieval-first machine-learning layer then ranks these triggers by their similarity, in this interpretable feature space, to a small seed catalogue of independently confirmed events, within an iterative human-in-the-loop process that introduces local supervised noise-rejection gates only for recurrent artefact families once they have been labelled. Applied to the CO2CRC Otway Stage 4 dataset—120 days of recordings on two wells, comprising roughly 23 TB and 14.14 million raw triggers—the workflow expanded a 39-event seed catalogue into 631 analyst-reviewed events, demonstrating complementarity with an independent template-matching analysis: two additional induced-event candidates were recovered, one within the CRC4 template-matching coverage and one on CRC7 during a CRC4 data gap. The induced-event class itself grew only from four to six candidates, and its counts are reported as a reviewed lower bound rather than a complete census. The result is a provenance-preserving, conservatively interpreted event inventory rather than an opaque classifier output, an outcome aligned with the reproducibility and audit requirements of CO_2_ storage assurance.

## 1. Introduction

Geological storage of CO_2_ requires monitoring systems that can provide assurance of containment, detect unexpected geomechanical responses, and remain practical over long operating periods [1,2,3]. Seismic monitoring is central to this task because it can image plume evolution, constrain reservoir response, and provide evidence for storage conformance. At the same time, monitoring programmes are increasingly expected to operate with lower surface impact, higher temporal repeatability, and stronger automation than conventional campaign-style surveys. The CO2CRC Otway International Test Centre in Victoria, Australia, has become an important field laboratory for developing and validating such monitoring technologies under controlled injection conditions [4,5,6].

The Otway programme has progressively tested active, passive, borehole, surface, and fibre-optic monitoring approaches. Stage 3 demonstrated monitoring of a small CO_2_ injection using a multi-well DAS array and permanent seismic sources, enabling high-repeatability time-lapse vertical seismic profiling during injection [7,8,9]. Subsequent studies extended this concept by using earthquake-generated wavefields and DAS-based passive seismic recordings to monitor changes and seismic response associated with injected CO_2_ [10,11]. Stage 4 continues this trajectory through continuous multi-well DAS monitoring of a small-scale CO_2_ injection conducted from 10 November 2024 to 21 January 2025, targeting an interval approximately 50 m above the Stage 3 injection interval [12].

DAS is particularly attractive for storage monitoring because a single fibre can act as a dense seismic sensor array, providing high spatial sampling along a borehole or cable over long monitoring periods [13,14,15]. DAS has been used across a growing range of geophysical applications, including vertical seismic profiling, earthquake monitoring, microseismic monitoring, near-surface imaging, volcanic monitoring, urban and infrastructure-scale sensing, and submarine-cable sensing [14,16,17]. For CO_2_ storage, downhole DAS is especially valuable because it can combine repeatable active-source monitoring with passive recording on the same installed fibre system [7,10,11,12]. However, the same dense sampling that makes DAS attractive also creates a processing challenge: continuous recordings contain large data volumes, variable coupling and noise conditions, and multiple classes of coherent signals that are not all related to injection-induced seismicity [14,15,18].

Passive seismic monitoring is an important complement to active time-lapse imaging because it can provide continuous information about induced seismicity, regional earthquake arrivals recorded by the DAS array, and other coherent wavefield phenomena without requiring repeated active-source surveys. In CO_2_ storage, induced seismicity is relevant both as a risk-management concern and as a possible indicator of pressure diffusion, fault activation, or geomechanical deformation associated with injection [19,20]. In practice, however, passive DAS monitoring does not produce a catalogue directly. It produces continuous sensor-network data from which candidate events must be detected, filtered, interpreted, and validated.

Event detection in continuous seismic data has traditionally relied on amplitude-based triggering, especially short-term-average/long-term-average (STA/LTA) methods, because they are simple, computationally efficient, and effective for abrupt energetic arrivals [21,22]. For DAS arrays, many events of interest are not defined only by local amplitude increase; they are expressed as coherent moveout across neighbouring channels. This has motivated spatial-coherence and semblance-based detectors that exploit the dense spatial sampling of DAS [18]. In this study, STA/LTA and semblance detection are treated as complementary trigger families within a common screening framework: STA/LTA provides an amplitude-based baseline, while semblance emphasises DAS-specific spatial coherence and moveout.

A second challenge appears after detection: detector triggers do not necessarily correspond to a genuine signal of interest. The task is therefore to attribute each trigger to its source, distinguishing among induced-event candidates, regional earthquakes, ocean-generated wavefields, surface activity, cable-related noise, instrumental artefacts, or ambiguous transient patterns. This distinction is operationally important because raw trigger counts can exceed the reviewable event population by many orders of magnitude. In the Otway Stage 4 analysis considered here, detector streams from continuous DAS data acquired in two wells produced 14.14 million raw triggers over 120 days. Managing this population required a machine-learning layer that could convert raw detections into structured, comparable objects and prioritise the subset most likely to contain coherent seismic wavefields.

Machine learning has increasingly been used to automate or accelerate DAS event detection, event localisation, denoising, and phase picking [23,24,25,26]. Supervised deep-learning approaches are well suited when the target event population is represented by a sufficiently large labelled field catalogue or when synthetic training data can be validated against substantial independent field evidence. Early-stage passive monitoring of CO_2_ storage is often different. Confirmed induced events may be rare, and the negative class (all non-induced signals) is difficult to define—not because negative labels are merely absent at the outset, but because that class is open-ended and heterogeneous across millions of triggers. Moreover, the absence of detections by a trained classifier cannot be interpreted as evidence that no coherent seismic events occurred. In this setting, the main limitation is not simply the ability to train a model, but the ability to evaluate its completeness, false-negative behaviour, and robustness to previously unseen noise conditions, acquisition geometries, and operational environments. These constraints argue not against supervised learning, but against deploying it as the first and only stage while labels are still scarce. What remains less developed is a transparent way to triage these very large trigger populations under sparse labels: to rank candidates for analyst review while preserving the provenance that later audit and reprocessing require.

This motivates a retrieval-first formulation rather than a fully supervised event-classification formulation imposed from the outset. In conventional seismology, waveform similarity and template matching have been highly effective for detecting repeated or nearly co-located events when representative master waveforms are available [27,28,29]. However, template matching and retrieval-based catalogue expansion answer different operational questions. Template matching is intentionally sensitive to such near-repeats—signals that share source, path, and recording response with a known waveform. Retrieval-based catalogue expansion, by contrast, is used here to prioritise coherent DAS patterns for analyst review across a heterogeneous trigger population. In the Otway Stage 4 case, the trigger population spans multiple signal types, with no single master waveform that could represent it. The proposed Argus DAS workflow therefore uses sparse analyst-confirmed labels as seed examples for retrieval, not as exhaustive class labels or fixed waveform templates. Supervision is staged rather than excluded. Each round of analyst review enlarges the labelled catalogue, and supervised components are added only where the labels make them well-posed: first, local gates that reject recurrent noise families with enough examples and later—if labels allow—optional stronger classifiers. Even then, these classifiers act as an additional noise-rejection layer within the labelled regime they cover, not as a global event detector over the full unlabelled trigger population.

Each trigger is represented as a two-dimensional time–channel DAS pattern and described by interpretable geometric, waveform, mask-shape, coherence, spectral, background, and contextual attributes. These attributes are transformed, robustly scaled, and screened for task-specific discriminability using the available seed labels so that proximity in the resulting feature space reflects similarity in abstracted coherent-wavefield character rather than waveform-level repetition.

The objective is not to declare all visually similar patterns as microseismic events, nor to replace human interpretation with an opaque classifier. The objective is to concentrate analyst review on the parts of a very large trigger population most likely to contain physically meaningful seismic signals, while retaining enough provenance for audit, reprocessing, and industrial decision support. External templates or pre-trained detectors from other DAS deployments may provide useful starting points in some settings, but they cannot remove the need for site-specific validation because DAS records strain along the fibre and the recorded response depends on cable geometry and coupling, gauge length, interrogator response, local velocity structure, and noise conditions [14,15]. The proposed approach therefore treats machine learning as a provenance-preserving triage and catalogue-expansion layer rather than as a stand-alone event detector.

This paper presents the Argus DAS sparse-label machine-learning workflow and its application to the CO2CRC Otway Stage 4 passive-seismic monitoring dataset. The contribution is threefold. First, we implement a scalable, auditable workflow for converting continuous multi-well DAS recordings from a CO_2_ storage experiment into a reproducible event-candidate catalogue. Second, we develop an interpretable machine-learning representation in which feature transformation, robust scaling, task-specific discriminability screening, high-recall cluster gating, retrieval-style ranking, and progressively introduced supervised noise gates are combined to prioritise candidates under extreme class imbalance. Third, we evaluate the workflow on an operational field dataset and show how a small seed catalogue of independently confirmed events can be expanded into a broader event inventory while preserving the provenance needed for scientific audit and future reprocessing.

## 2. Materials and Methods

### 2.1. Dataset and Monitoring Objective

The dataset analysed in this study comprises continuous distributed acoustic sensing (DAS) recordings from the CO2CRC Otway Stage 4 monitoring programme. The analysed window covers 120 days from 1 November 2024 to 1 March 2025, spanning pre-injection baseline, the Stage 4 CO_2_ injection period (10 November 2024 to 21 January 2025), and post-injection monitoring. Passive screening was applied to representative downhole DAS intervals from wells CRC4 and CRC7, which provided good data quality and spatial coverage of the monitored interval.

**Figure 1:**
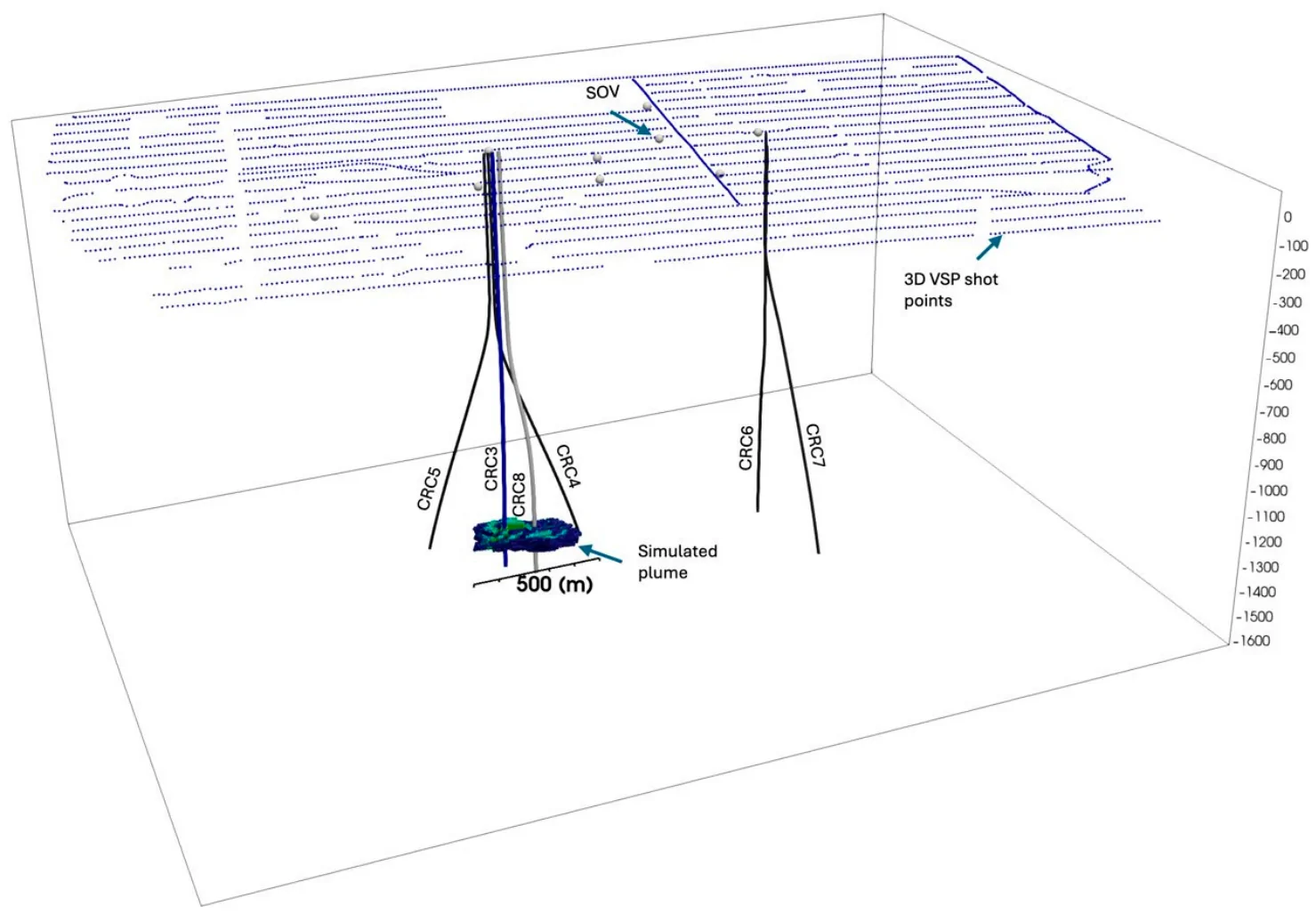
Acquisition geometry of the CO2CRC Otway Stage 4 monitoring programme: downhole fibre-optic instrumented wells (CRC3–CRC8), surface orbital vibrators (SOVs), and 4D vertical-seismic-profiling shot points, with the simulated CO_2_ plume at the injection interval. The passive-screening analysis of this study uses the CRC4 and CRC7 wells. Reproduced from Pevzner et al. [12].

The Otway Stage 4 programme instruments a multi-well downhole fibre-optic array (wells CRC3–CRC8) together with a set of continuously operating surface orbital vibrators (SOVs) that provide repeated active-source excitation, complemented by a sparse 4D vertical-seismic-profiling component; the acquisition geometry is shown in Figure 1 [12]. The present passive-screening study uses two of these wells, CRC4 and CRC7, each interrogated with a Silixa iDAS v3 unit (Silixa Ltd., Elstree, UK) on an engineered fibre cemented behind casing, with a 10 m gauge length [12]. Continuous multi-unit recording generates on the order of 6 TB of raw data per day, reduced on site by an edge-computing system; the decimated SEGY archive analysed here samples each well at 500 Hz with a channel spacing of approximately 5.1 m (Table 1). Full details of the Stage 4 acquisition design, instrumentation, and on-site data handling are given by Pevzner et al. [12].

Raw recordings from the Silixa iDAS interrogator (DAS strain-rate) were converted into a decimated SEGY archive that served as the canonical input to the workflow. For this analysis, each well was represented as a two-dimensional time–channel matrix sampled at 500 Hz with a nominal channel spacing of approximately 5.1 m. The CRC4 and CRC7 analysis intervals contained 322 and 319 channels, respectively, corresponding to a downhole sensing aperture of about 1.6 km in each well (Table 1).

Performing this screening over a multi-month, multi-well DAS archive is itself a systems problem rather than a modelling one. Before any event can be prioritised, the continuous recordings must first be turned into a population of well-defined candidate objects that a model can compare. This study therefore builds two coordinated parts: a deterministic front end that reads the raw archive reproducibly and converts it into structured trigger candidates with precomputed attributes (Section 2.2) and a sparse-label machine-learning layer that ranks those candidates for analyst review and catalogue expansion (Section 2.3).

### 2.2. From Continuous DAS Data to Trigger Objects and Features

**Figure 2:**
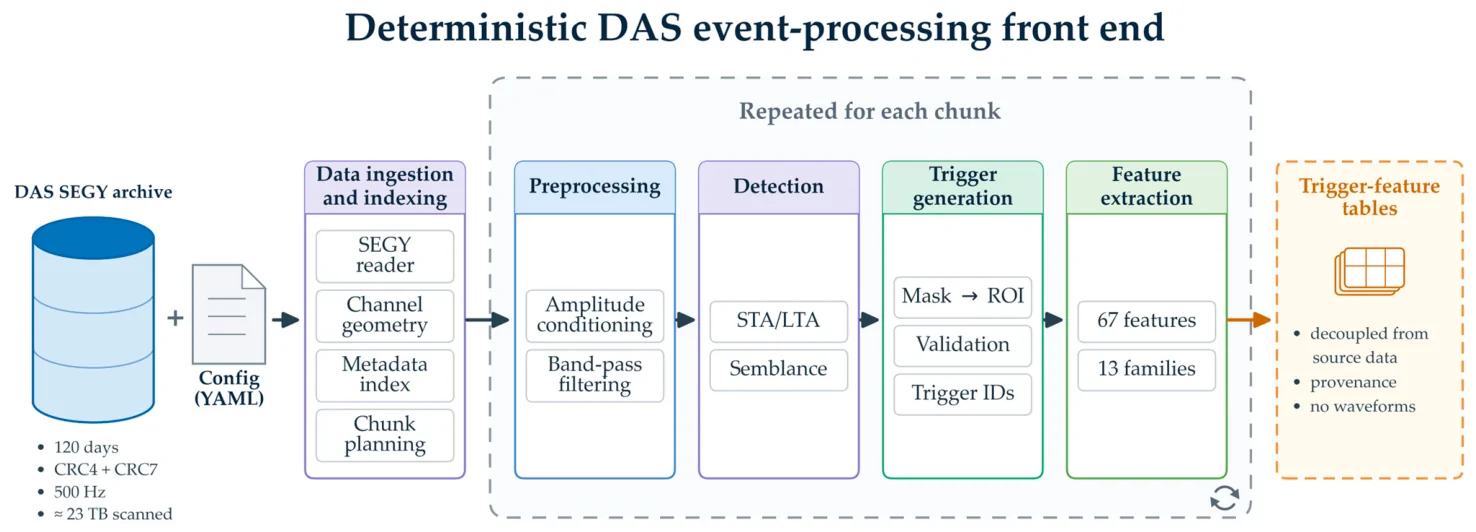
Deterministic front-end workflow used to convert continuous DAS recordings into trigger-level feature tables.

Before any machine-learning prioritisation, the continuous DAS archive must be converted into a structured population of comparable candidate objects. In Argus, this is done by a deterministic, configuration-driven front end, implemented in Python (version 3.12), that operates chunk-by-chunk over the DAS SEGY archive (Figure 2). Its purpose is not to classify events, but to manufacture reproducible trigger objects with well-defined time bounds, channel bounds, detector provenance, and interpretable attributes. This separation matters because continuous DAS archives reach terabyte scales (about 23 TB in this study), confirmed seismic events within them are sparse, and the same multi-month record may need to be reprocessed as detector settings, labels, or monitoring objectives evolve.

The workflow began by converting the DAS SEGY archive into a standard time–channel representation suitable for detector execution. SEGY files were read through a dedicated input layer that separated data access from the downstream processing logic, while geometry and timing metadata were attached separately, keeping the processing logic independent of the storage format. The multi-month archive was then indexed by time, channel coverage, and continuous recording segments so that processing could be planned directly from the physical extent of the data rather than from individual files. This index-driven design allowed the archive to be divided into reproducible chunks—each chunk spanning up to two hours of continuous recording across all analysed channels of a well (of order 3.6 million time samples × ~320 channels at 500 Hz), processed with short (60 s) filter-warmup margins—with recording gaps excluded and each chunk treated as a comparable unit for subsequent preprocessing, detection, and feature extraction; the 120-day record was divided into 5354 such chunks (Section 3.1).

Within each chunk, a fixed two-stage preprocessing sequence was applied before detection. First, robust per-channel amplitude clipping (median/MAD-based) removed extreme instrument spikes before they could trigger the energy-based detectors. Second, an Ormsby band-pass filter (10–20–100–240 Hz) restricted the data to the microseismic signal band, suppressing out-of-band noise. The same configuration was used for every chunk, and the applied filter parameters were stored with each resulting trigger for reproducibility.

Two complementary detector families were applied to each preprocessed chunk. STA/LTA [21,22] was used to capture abrupt amplitude increases, whereas a semblance-based detector [18] was used to emphasise spatially coherent arrivals with moveout across neighbouring DAS channels. These detectors therefore view the same DAS record from different perspectives: one highlights amplitude anomalies, while the other highlights coherent two-dimensional time–channel patterns. Running both streams improves recall and makes it possible to retain candidates that may be weak in amplitude but organised in space.

Detector output was not treated as a final event catalogue. Each detector produced candidate regions in time–channel space, which were grouped into trigger objects with finite duration, channel extent, and spatial structure. The framework supports configurable geometric plausibility filters—minimum duration, channel span, and spatial fill—that can pre-screen triggers according to the expected size and shape of the target events. In this study, these filters were set permissively (minimum duration of 30 ms and channel span of about 100 m) so that even small or low-amplitude arrivals were retained, and the remaining thresholds were kept deliberately loose so that weak but spatially coherent candidates were preserved for later ML-assisted review. The complete production front-end parameter set is listed in Appendix A.

Each trigger stores only the information required to locate, compare, and review the candidate: its time and channel bounds, a representative centre point (time and channel), mask geometry, and processing provenance. Rather than duplicating the waveform, the trigger acts as a compact reference that points back into the DAS record; feature extraction then reads the actual waveform samples within this footprint to compute interpretable attributes, which in turn support quality control, machine-learning ranking, and catalogue review. In this way, the deterministic front end reduces the continuous multi-month DAS archive to a reproducible population of comparable trigger objects, separating field-scale signal screening from sparse-label machine-learning prioritisation.

#### Interpretable Feature Representation

Each trigger is first treated as a localised two-dimensional DAS panel: a candidate pattern in time and channel that an analyst could inspect directly. The workflow describes this pattern using observable characteristics—its duration and channel (depth) extent, detector-mask shape, apparent moveout across channels, amplitude, and signal-to-noise behaviour, spectral content, surrounding background noise, and temporal context within the detector stream. Computed while the waveform samples are still in memory, these descriptors are assembled into a fixed 67-feature vector organised into 13 semantic families (Table 2; each feature is defined in Appendix B, Table A1). This vector is the numerical representation consumed by the machine-learning stage, rather than the raw image itself. Because each feature has a clear physical or operational meaning and remains linked to its source trigger, proximity in this representation can be interpreted as similarity in abstracted DAS wavefield character rather than waveform-level repetition. This feature bank is deliberately built as a set of interpretable axes of visual and physical variation—compact versus extended, filled versus fragmented, coherent versus poorly repeated, impulsive versus sustained, isolated versus burst-like—and not as predefined event labels; class interpretation enters only later, through sparse analyst-assigned labels and task-specific feature screening. This preference for interpretable representations over opaque end-to-end models in review-critical applications follows the argument of Rudin [30].

This bank holds 67 features in total, and its size is a deliberate design decision. Feature extraction is the only expensive point of contact with the raw archive: once a trigger is reduced to its feature row, downstream machine learning never revisits terabytes of SEGY, but rerunning extraction to add or edit individual features is impractical. We therefore compute a broad, stable attribute bank once and treat it as a reusable, hand-designed embedding of each trigger. Because we do not know in advance which attributes will matter, different tasks draw on different subspaces of this embedding: each trigger becomes a point whose proximity to others reflects similarity in wavefield character, and the geometry is re-posed per task—high-recall rejection of background clutter, binary target-versus-noise separation, filtering of a specific artefact family such as orbital or common-mode array noise, and finer inter-class separation as labels accumulate—without recomputation. Which subspace is used for a given task is selected by the discriminability screening described in Section 2.3.2.

### 2.3. Sparse-Label Machine-Learning Workflow

The machine-learning layer is not a trained event classifier but an iterative, human-in-the-loop similarity search whose job is to decide where an analyst should look first. It starts from what is actually available early in a monitoring programme: a small seed catalogue of confirmed events—here assembled from independent screening workflows applied to the same data (Section 2.4)—set against millions of unlabelled detector triggers and, initially, no labelled noise at all. In machine-learning terms this is a positive–unlabelled learning problem [31], in which supervised training and, more importantly, supervised evaluation remain ill-posed until reliable negative labels accumulate. Instead of asking a model to assign every trigger to a class, the workflow treats the labelled events as queries and retrieves the unlabelled triggers most similar to them in the interpretable feature space of Section 2.2. Because that space is built from physical and geometric attributes rather than raw waveforms, similarity reflects shared wavefield character—duration, moveout, coherence, spectral shape—so the search generalises across events of the same kind without requiring waveform-level repetition.

**Figure 3:**
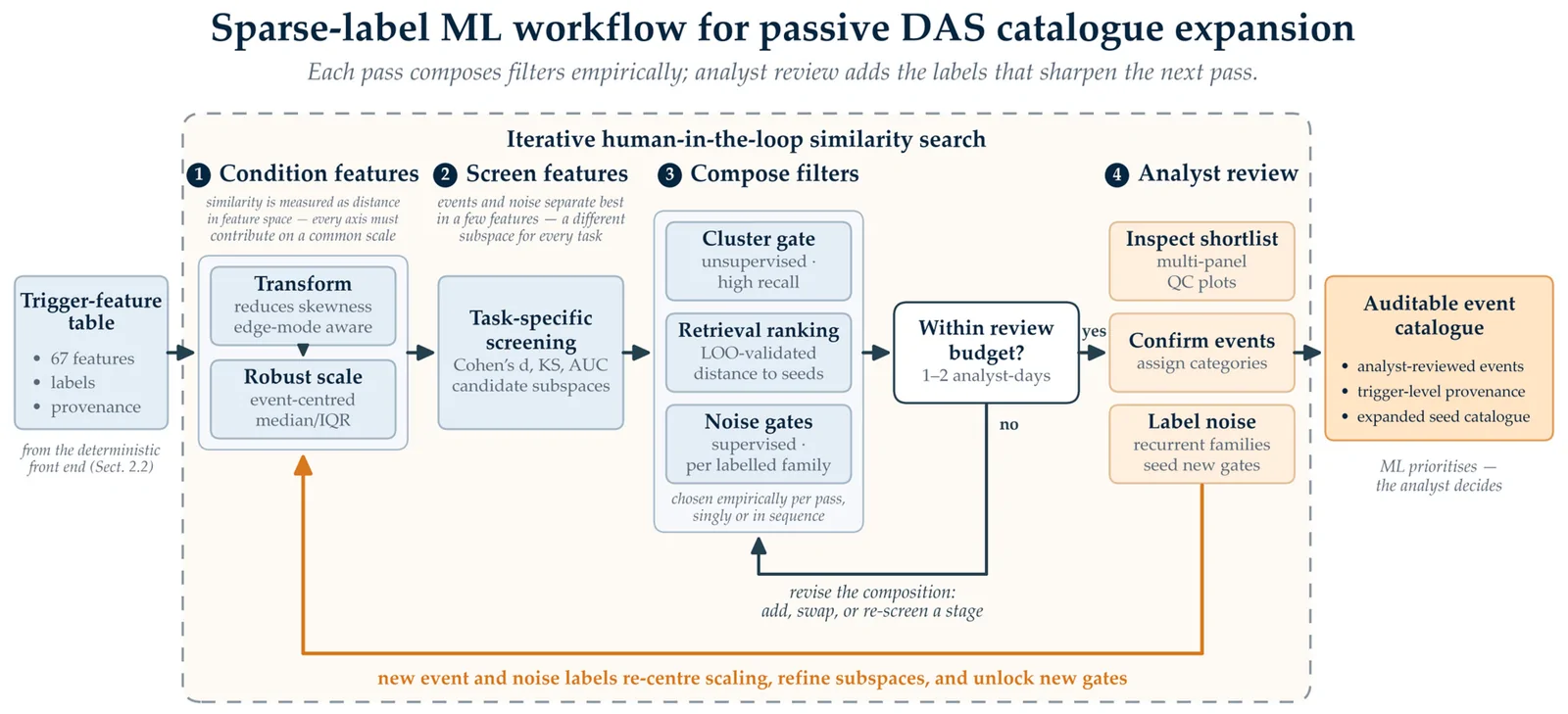
Sparse-label machine-learning workflow: an iterative human-in-the-loop similarity search in which empirically composed filter passes reduce millions of detector triggers to a ranked review set, and analyst review returns the event and noise labels that sharpen the next pass.

A single pass through the workflow (Figure 3) is deliberately simple: features are conditioned for distance computation (Section 2.3.1), screened for the subspace most discriminative for the task at hand (Section 2.3.2), and passed through a short composition of filtering stages drawn from a toolbox of three complementary families (Section 2.3.3)—an unsupervised high-recall cluster gate, retrieval-style ranking against the seed events and, once the corresponding labels exist, supervised gates that reject recognised noise families. What is not fixed is the composition itself. No single algorithm can be guaranteed in advance to compress millions of heterogeneous triggers into a reliable shortlist, so which stages are applied, in which order, and in which feature subspace is an empirical decision made anew at each pass. A candidate composition is run end-to-end and judged against an operational criterion: the retained population must form a ranked review set small enough for one analyst to inspect within one to two days. If the retained candidates are still too numerous or are visibly dominated by a single recurrent artefact family, the composition is revised—a stage is added, swapped, or re-screened—before any analyst time is spent (inner loop in Figure 3).

Analyst review closes each pass and grows the toolbox along with the catalogue (outer loop in Figure 3). The analyst inspects multi-panel quality-control plots for the shortlisted candidates, confirms events, and—just as importantly—names any recurrent noise pattern as a labelled category of its own; the orbital-pattern and common-mode array noise families at Otway entered the label database this way. Each new noise category unlocks a dedicated supervised rejection layer in the next pass, while every confirmed event sharpens the screening and the ranking. The three-cohort structure of the trigger population—confirmed events, labelled noise families, and an unlabelled majority—is therefore an outcome of the loop rather than a precondition: the workflow begins with seed events and an unlabelled pool, and accumulates its negative classes opportunistically, one filtering task at a time. The unlabelled majority is never exhaustively labelled: it remains a heterogeneous pool that may contain both unrecognised noise families and, in principle, undiscovered events, so the labelled noise categories never amount to a complete model of the negative class. Throughout, machine learning only prioritises; final event identity and acceptance remain a human decision. The loop is thus a human-in-the-loop learning system [32,33]: each pass routes a small, deliberately selected batch of unlabelled candidates to the analyst for labelling—selected here by similarity to confirmed events rather than by model uncertainty, a strategy related to but distinct from classical active learning [32]—and the returned labels directly improve the next pass.

Successive passes do not re-examine triggers that have already been reviewed. Every trigger carries a persistent identifier, and from the second pass onward any trigger inspected in an earlier pass is excluded from the new review set, so each pass spends analyst time only on candidates that a filter has surfaced for the first time. The full trigger population is nonetheless retained throughout: all triggers, reviewed or not, continue to inform the feature transformation, the discriminability screening, and the filter parameters on every pass. Deduplication therefore applies to the manual review queue, not to the statistics that drive the search.

#### 2.3.1. Feature Conditioning for Distance-Based Learning

All per-chunk feature tables and labelled event catalogues are first aggregated into a single analytical database. Labelled catalogues and production-run triggers are loaded as independent groups, and cross-group deduplication is deferred so that catalogue labels can supersede run triggers without modifying immutable run data. Feature transformation then prepares all attributes for distance computation: per-feature edge modes are detected, candidate transformations (identity, logarithmic, square-root, logit, Yeo-Johnson [34]) are evaluated on the distributional core, and adaptive density clipping limits extreme tails while protecting labelled events.

Scaling maps each transformed feature into a dimensionless z-space using a median centre and an interquartile-range scale computed from the labelled seismic events rather than from the full background population. This event-centred choice is deliberate: it makes one unit of distance correspond to one robust spread of the event population on every axis so that proximity is weighted around the signals being searched for instead of being dictated by millions of background triggers.

#### 2.3.2. Task-Specific Screening and Subspace Generation

Feature screening is repeated inside each downstream task rather than performed once globally, because every pass asks a different question of the same 67-feature representation. For the population pair relevant to the task—target versus unlabelled triggers when rejecting background clutter, target versus one labelled noise family when building a dedicated gate—the workflow computes Cohen’s d [35], the Kolmogorov–Smirnov (KS) statistic [36], and AUC-ROC [37] for each feature and combines them into a composite discriminability score, computed as

*s_f_* = (1/|*M_f_*|) *Σ_m_*_∈_*_Mf_* (*x_m,f_* − *min_g_ x_m,g_*)/(*max_g_ x_m,g_* − *min_g_ x_m,g_*) (1)

where *x_m_*_,*f*_ is the value of metric *m* for feature *f*, with the metrics being |Cohen’s d|, the Kolmogorov–Smirnov statistic, and the direction-invariant AUC-ROC, max(AUC, 1 – AUC); the minima and maxima are taken over all candidate features *g* of the task; and *M_f_* is the set of metrics available for feature *f* (all three in the standard case); a metric whose values coincide across all candidate features contributes zero. Features are ranked by *s_f_* in descending order. The feature subspaces this procedure selected for the gates of each stream’s final filter composition (Section 2.3.4) are listed in Appendix C.

Candidate subspaces are then generated by several strategies, including composite-score-ranked, single-metric-ranked (e.g., KS-only), correlation-pruned, orthogonal, group-balanced, and constrained-random selection. The feature set that best strips background clutter is rarely the one that best separates target events from a specific artefact class, and because the labelled populations change after every review round, screening is rerun whenever they grow. The subspaces therefore adapt automatically to the current state of the labelled catalogue.

#### 2.3.3. A Toolbox of Complementary Filters

The first family is an unsupervised, high-recall cluster gate. K-means clusterings [38] (scikit-learn, version 1.8.0) are swept over many candidate subspaces and cluster counts, and the workflow then searches for a compact subset of clusters that contains the labelled target events while minimising the number of unlabelled triggers retained. K-means is preferred over more elaborate clustering algorithms deliberately: after the transformation and robust scaling of Section 2.3.1, every screened subspace is a standardised space in which Euclidean distance is the intended measure of similarity, so a simple centroid-based partition matches the geometry, scales comfortably to millions of triggers, and keeps the gate transparent enough to audit. The search is governed by retention principles rather than fixed numerical thresholds. An aggregate recall floor on the labelled event family is set by the analyst for each pass: it can be held at 100% while labelled events are few and relaxed as the catalogue grows and acquires weak or atypical members whose loss at this stage would be tolerable. Independently of the aggregate floor, a premium subset of labels—induced-event candidates in this study—must be retained in full, and a margin-safety criterion rejects configurations that place premium events close to the boundary of a discarded cluster even when no event is actually lost. Within these constraints, the gate simply minimises the number of unlabelled triggers passed forward. The design is deliberately conservative: passing extra candidates to review is acceptable, whereas losing a scarce induced-event example at the first machine-learning stage is not.

The second family is retrieval-style similarity ranking, which requires no negative labels at all. The labelled events are condensed into geometric prototypes in the screened subspace—centroids, class-balanced or class-weighted centroids computed across the different labelled event classes so that the prototype emphasises properties shared by coherent seismic arrivals rather than the features peculiar to whichever class is most numerous, or k-nearest-neighbour references—and every unlabelled trigger is scored by its distance to the prototype. Configurations are selected by strict leave-one-out validation: each labelled event is held out in turn, the prototype is rebuilt without it, and the held-out event is ranked against the unlabelled pool. A combination of subspace, distance metric, and prototype construction that consistently places held-out events near the top of millions of unlabelled candidates is then applied to the full population, and the top of its ranked list becomes the review set. This criterion is a working hypothesis rather than a guarantee: it verifies that known events of different classes concentrate in a compact region of the chosen subspace and assumes that unlabelled triggers falling in the same region are the candidates most worth inspecting first. Under extreme class imbalance, the assumption cannot be proven in advance; it is tested operationally by whether each review pass keeps confirming new events near the top of the ranked list.

The third family is supervised rejection of recognised noise, and it exists only where review has already produced negative labels. Cascaded category-specific gates each target one labelled noise family—such as orbital-pattern noise, common-mode array noise (a near-simultaneous activation of all or most channels along the cable, appearing as array-wide ringing and most plausibly reflecting a mechanical disturbance at the instrument), or surface noise—with their own screening and explicit per-label loss budgets so that removing noise cannot silently consume scarce target events. Each gate is screened for the features that separate its noise family from the entire labelled event family pooled across classes, which forces the gate to exploit contrasts that hold against every kind of confirmed event rather than against an average one. The distinction matters because the boundary is not simply coherent signal versus incoherent noise: orbital patterns, for example, are themselves coherent seismic wavefields—the signature of the surface orbital vibrators (SOVs) operating continuously at the site for active-source monitoring [12]—and lie close to genuine events along many feature axes. Once enough labelled noise has accumulated for stable cross-validation, the category-specific cascade can be supplemented—only within the labelled, reviewed regime—by a single binary classifier based on gradient-boosted decision trees (LightGBM, version 4.6.0) [39], trained to separate the labelled events from all labelled noise at once; its rejection threshold is chosen on held-out labels so that even in the worst validation split essentially no confirmed events are sacrificed. This classifier acts as an additional operational rejection layer for recognised noise, not as a global event/noise detector over the full unlabelled trigger population, and it is never used to declare the catalogue complete. Whether this third family is engaged at all is an operational decision. On CRC4, the first two families still left a review set that was both large and visibly dominated by recurrent noise patterns, so labelling those patterns and adding dedicated gates was the productive next step. On CRC7, where only the semblance stream entered ML-assisted expansion (Section 3.1), the first two families alone reduced it to a population manageable by direct review, and the surviving candidates went straight to the analyst—exactly the regime a newly opened monitoring deployment would face.

#### 2.3.4. Iterative Composition and Analyst Review

The workflow is therefore an empirical search procedure operating under two fixed constraints, rather than a fixed pipeline: scarce high-value labels must never be lost, which is enforced inside every stage as recall floors, margin safety, and loss budgets; and the review set must never exceed what the analyst can absorb. Within those constraints, iteration is cheap and encouraged. A composition that leaves too many survivors is extended with a further stage; one whose shortlist is dominated by a recognisable artefact motivates labelling that artefact and adding a gate for it; one that performs well is kept and simply rerun as the labels grow. Noise is labelled only when a recurrent pattern threatens the review budget, not in an attempt to enumerate the negative class exhaustively, so the labelled noise families remain a working set of recognised artefacts rather than a complete model of everything the unlabelled pool may contain. At Otway, the loop evolved in exactly this way: early passes relied on the cluster gate and similarity ranking alone, and the supervised noise gates emerged later, one labelled family at a time.

Analyst review closes the loop. For every shortlisted candidate the workflow generates multi-panel quality-control plots—the band-passed DAS panel, the detector mask, and the detector characteristic functions, i.e., the running detection statistics (the STA/LTA ratio and the semblance value) that are thresholded to produce the binary detection masks—and the analyst assigns the final category and acceptance status. What this step requires of the human reviewer is consistent visual classification of recurring time–channel patterns against the QC panels. Because every decision is written back against a persistent trigger identifier, individual judgements remain auditable and can be revisited as the catalogue matures. Each accepted event remains linked to its trigger identifier, detector stream, feature row, processing configuration, and source data, so the catalogue that accumulates is an analyst-reviewed scientific product, with machine learning serving as an auditable prioritisation layer. Iterations continue while review keeps returning new confirmed events, and the design choices that keep this loop defensible under sparse labels are summarised in Table 3.

### 2.4. Seed Event Catalogue

The sparse-label workflow of Section 2.3 is initialised by a seed catalogue of confirmed events. The seed events used in this study were identified independently of the Argus pipeline by two complementary screening efforts within the project team and were adopted as the initial labelled examples for scaling, screening, ranking, and gating. The first source was an in-house travel-time-based screening tool, which scans the decimated SEGY archive for arrivals consistent with predefined travel-time curves [11] and renders each candidate as a time–channel image panel; these panels were visually classified into induced, regional earthquake, ocean-generated, and strong surface-related examples. This tool was the sole source of the regional earthquake, ocean-generated, and strong surface-related seed events and also surfaced induced candidates. The second source was an independent 2D template-matching analysis of the raw (undecimated) CRC4 recordings (method of [40]), focused specifically on induced microseismicity; it supplied the four induced-event candidates in the seed catalogue, several of which were independently flagged by the in-house tool as well.

The assembled seed catalogue comprised 39 distinct events—4 induced-event candidates, 11 regional earthquakes, 7 ocean-generated events, and 17 strong surface-related events—represented by 153 labelled triggers in the feature database, since one physical event can produce triggers on both wells and from both detector families (Section 2.5). Two properties of this seed set shaped the design choices of Section 2.3: it is far too small to train a conventional event classifier, and its scarcest class, the induced events, is also its most valuable one. Because the seed events were produced by detection methods independent of this workflow, they also serve as an external reference point: Section 3 compares the ML-expanded catalogue against the template-matching analysis on the induced-event class.

The composition of the seed set also fixed a central design decision of the workflow. With only four induced-event examples, a search for further events of that class alone would rest on support too small to define similarity reliably in any screened subspace. The workflow therefore pools all confirmed classes—induced, regional earthquake, ocean-generated, and strong surface-related events—into a single labelled family of coherent seismic wavefields for the purposes of scaling (Section 2.3.1), screening (Section 2.3.2), and gating (Section 2.3.3). The grouping is physically motivated rather than merely pragmatic: despite their very different sources and scales, all four classes are elastic wavefields propagating across the array, and on the interpretable axes of Section 2.2, they share the signatures that separate organised wavefields from clutter—coherent moveout, spatial continuity of the detector mask, and structured spectral content. Pooling raises the effective seed support from 4 events to 39, while the class-balanced prototypes of Section 2.3.3 keep the numerous surface class from dominating the pooled geometry and the premium-label protections of the cluster gate keep the induced examples from being traded away. The assumption that the classes occupy a common region of feature space is treated as a working hypothesis rather than a premise: it is exactly what the leave-one-out validation of Section 2.3.3 tests on every pass, and Section 3.2 shows it directly in a projection of the gated feature space.

### 2.5. Event-Level Catalogue Assembly and Evaluation

The detector and ML stages operate on triggers, not directly on physical events. A single physical signal may be detected on different wells, by different detector families, or as several neighbouring trigger fragments. For reporting, validated triggers are merged into event-level records using temporal coincidence: triggers are sorted by start time and merged greedily, a trigger joining the current event if its start lies within a category-specific merge gap of the latest end time among the triggers already grouped, across both wells and both detector families. The merge gap is 2 s for induced and ocean-generated events, 3 s for strong surface-related events, and 45 s for regional earthquakes, whose P and S arrivals and coda span tens of seconds; the running-latest-end rule also absorbs the inter-well arrival delay (typically 30–450 ms for induced events). Event windows are defined from the participating triggers, and per-well status is recorded as detected, not detected, or no data.

Evaluation is therefore framed operationally rather than as a conventional supervised-classification benchmark. The main quantities are the scale of the raw trigger population, the size and composition of the labelled seed catalogue, the expansion of the event-level catalogue after ML-assisted review, the detector streams contributing to accepted events, and the per-well detectability pattern. These metrics are appropriate for sparse-label field monitoring because they measure whether the workflow produces a reviewable, auditable catalogue without claiming completeness that cannot be independently verified. Section 3 reports these quantities for the Otway Stage 4 dataset.

## 3. Results

This section reports the application of the workflow to the Otway Stage 4 dataset in the order of the processing chain: the raw trigger population and its feature representation (Section 3.1), the catalogue expansion achieved under sparse labels (Section 3.2), the contribution of the two detector families (Section 3.3), per-well detectability (Section 3.4), and the induced-event candidates, including a comparison with an independent template-matching analysis (Section 3.5).

### 3.1. Trigger Population and Processing Scale

The deterministic front end (Section 2.2) was executed over the full 120-day window for both wells as six production runs (three consecutive time phases per well), processing 5354 archive chunks under a single, unchanged configuration. Across the four well–detector streams, the detection layer produced 14,144,883 raw triggers (Table 4). Every trigger was reduced to its 67-feature representation at extraction time and aggregated into a single analytical feature database of 7.4 GB, which served as the sole input to the machine-learning workflow; the 23 TB SEGY archive was revisited only to render QC panels for shortlisted candidates. The six production runs completed in approximately 140 h of wall-clock time in total (about 94 s per archive chunk on average; five runs on a 32-core workstation and one on a 48-core Linux server), so the 240 well-days of two-well recordings (23 TB) were traversed in under six days of machine time, about 41 times faster than the summed recording duration; runs with higher trigger counts took proportionally longer, feature extraction being the dominant per-trigger cost.

Table 4 also records why this population must be triaged rather than reviewed. At one second per trigger, exhaustive inspection of the full raw detector output would amount to more than 160 days of uninterrupted viewing, whereas the labelled seed catalogue available at the outset comprised 153 triggers—an imbalance of roughly five orders of magnitude between the labelled examples and the raw trigger population. The streams are also strongly imbalanced among themselves: CRC7 STA/LTA alone contributed 10.2 million triggers, more than 70% of the total, and visual sampling showed this stream to be dominated by dense single-channel impulsive transients with no spatial organisation (Section 3.3). Because these triggers form wherever such transients momentarily coincide across channels, they fall essentially at random across the record and overwhelmingly mark noise rather than spatially organised arrivals; carrying them into the similarity search would enlarge the review set with noise rather than with candidate events, so the stream was set aside after quality control rather than passed to ML-assisted expansion, leaving 3.9 million triggers in the three active streams. This exclusion applies to the similarity search only: where a CRC7 STA/LTA trigger coincides in time with an event confirmed on another stream, it is still attached to that event during event-level assembly (Section 2.5), which is why a small number of CRC7 STA/LTA triggers appear in the catalogue tables of Section 3.3 and Section 3.4. Together, these scales define the operational problem that the workflow of Section 2.3 was built to solve.

### 3.2. Catalogue Expansion Under Sparse Labels

Starting from the 39 seed events of Section 2.4, the workflow was iterated over the three active streams (Section 3.1) as described in Section 2.3.4. Each pass composed a filter chain from the three families of Section 2.3.3, reduced the active trigger stream to a ranked review set of roughly 1000–5000 candidates—within the one-to-two-day review budget—and returned two kinds of labels: newly confirmed events, and newly named recurrent noise families. The orbital-pattern, common-mode array, surface-noise, and general-noise categories all entered the label database through these passes, progressively unlocking the supervised gates of Section 2.3.3 on the CRC4 streams, although only the semblance stream retained them in its final pass (Appendix C). In total, the campaign evaluated 19 filter compositions across the three active streams (five on the CRC4 semblance stream plus three iterations of its gradient-boosted binary classifier, five on CRC4 STA/LTA, and six on CRC7 semblance); the individual gates of the final-pass composition of each stream are listed in Appendix C (Table A2). Analyst review and labelling were interleaved with composition development throughout, and per-pass analyst time was not logged separately, so the one-to-two-day budget acted as an operational ceiling on the size of each review set rather than as a measured turnaround time.

The final catalogue contains 631 events across the four target classes, a 16.2-fold expansion of the seed set (Table 5). The expansion is strongly uneven across classes, a pattern consistent with the differing recurrence rates of the underlying wavefields, although class yields also reflect the seed composition and the regions of feature space prioritised for review (Section 4.6). Ocean-generated and strong surface-related wavefields recur throughout the monitoring window, so similarity retrieval finds them in large numbers (7 to 309 and 17 to 302 events, respectively). Induced events and regional earthquakes are expected to be rare over the 120-day window of a small-scale injection, and the catalogue grew only modestly for these classes (4 to 6 and 11 to 14). For the induced class in particular, growth was modest: of the two added candidates, one (28 November 2024) lies within the CRC4 template-matching coverage but was not among its detections, while the other (11 December 2024) was recovered on CRC7 during an interval when CRC4 had no data and so falls outside the scope of the CRC4-only template-matching comparison (Section 3.5). These additions are best read as reviewed lower-bound evidence of operational coverage rather than as a demonstration that feature-space retrieval outperforms template matching. The final catalogue is therefore a filtered event inventory whose class proportions follow the wavefield rather than a uniform amplification of every seed class, and its scarce-class counts should be read as a reviewed lower bound rather than a complete census.

**Figure 4:**
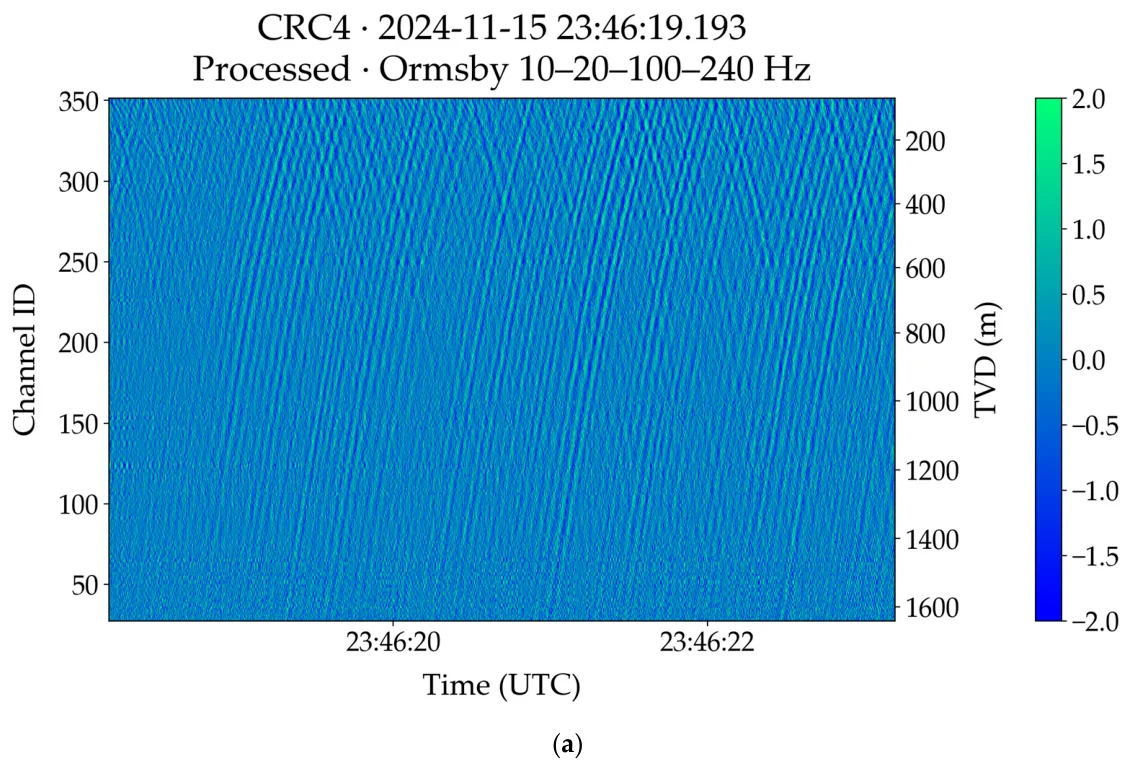

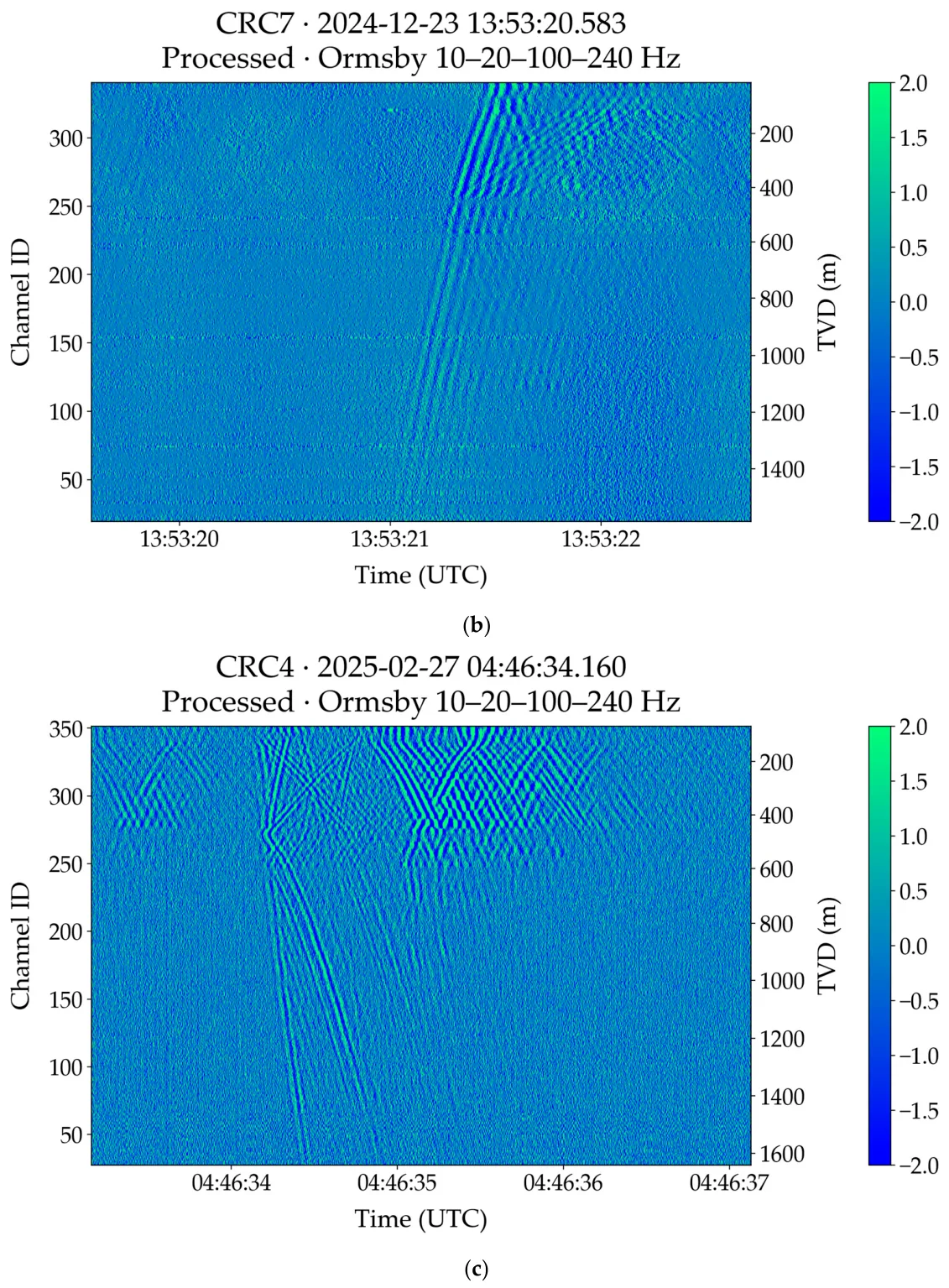
Representative events from the expanded catalogue classes, shown as band-passed time–channel DAS panels: (**a**) regional earthquake; (**b**) ocean-generated event; (**c**) strong surface-related event.

Throughout the expansion, no event entered the catalogue without visual confirmation: the machine-learning layer controlled only the order and the volume of what was reviewed, while every acceptance decision was made by the analyst on the multi-panel QC plots and recorded against the trigger identifier (Section 2.3.4). Figure 4 shows representative members of the three expanded non-induced classes as they appear in review.

**Figure 5:**
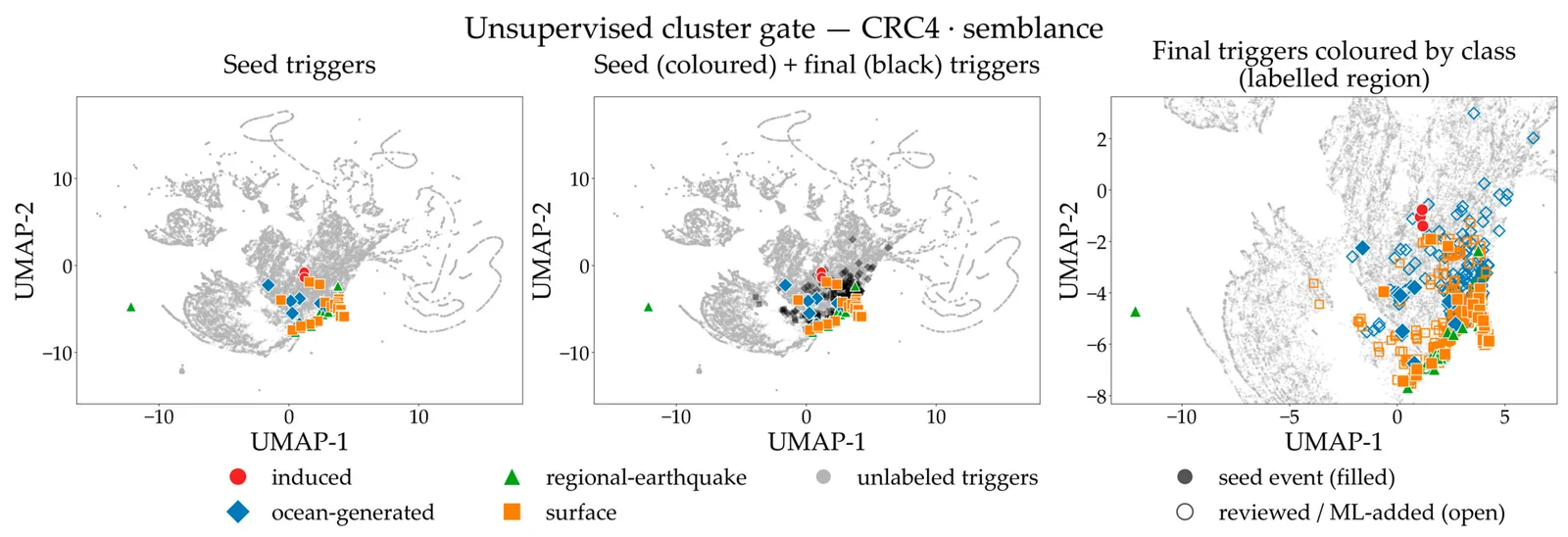
UMAP projection [41] of the four screened features (mask.triggered_channels, mask.coincidence_efficiency, mask.back_loaded_ratio, env.temporal_entropy) used by an illustrative seed-only unsupervised cluster gate on the CRC4 semblance stream (display only; the gate operates in the full four-feature space; the final-pass production subspace of this stream is given in Appendix C). Grey: unlabelled triggers. (**Left**): seed events coloured by class, concentrating in one compact region. (**Centre**): the same projection with the confirmed-catalogue triggers present in the CRC4 semblance stream (all workflow passes) added in black, filling the seed region and its surroundings. (**Right**): a zoom into the labelled region, with the reviewed (ML-added) triggers shown as open symbols coloured by class and the seed events as filled symbols so that the class composition of the triggers added during review can be read directly. The axes (UMAP-1, UMAP-2) carry no physical units; the layout indicates which triggers are neighbours but does not represent absolute distances or densities.

Figure 5 shows the geometry behind this expansion for one stream, the CRC4 semblance detector, as a UMAP projection (umap-learn, version 0.5.11) of the feature subspace the gate works in [41]. UMAP lays out each trigger as a point in the plane so that triggers with similar features end up near one another; the two axes carry no physical units, and the layout is read to determine which points are neighbours, not for exact distances, cluster sizes, or densities, none of which UMAP preserves reliably. The reduction here is gentle: the configuration shown here—an illustrative cluster gate selected on the seed labels alone by the procedure of Section 2.3.3—uses only four screened features: the number of triggered channels (mask.triggered_channels), how coincidentally they activate (mask.coincidence_efficiency), how the activation is distributed through the trigger duration (mask.back_loaded_ratio), and the energy-envelope entropy (env.temporal_entropy)—mask shape and timing rather than amplitude. The final-pass production gate on this stream used a ten-feature subspace selected by the same procedure. Appendix C lists the subspaces of the final-pass gates of every stream. The plane thus compresses four dimensions, not the full feature bank, and the neighbourhoods it shows stay close to those the gate actually sees.

In the left panel, only the seed events are coloured, over the grey unlabelled background, and the four classes—induced, regional earthquake, ocean-generated, and strong surface-related—fall into one compact region instead of spreading across the population. This grouping is not built in by the method: the feature screening of Section 2.3.2 separates events from unlabelled clutter, not one event class from another, so the classes are free to scatter and yet do not. Their sharing a region is the condition that the pooled-family choice of Section 2.4 depends on—it is what allows the four scarce induced examples to be searched for together with the other confirmed events rather than alone—and it holds across the screened subspaces examined during gate selection, more tightly in some and more loosely in others; the configuration shown was chosen only because it renders that shared region most clearly.

In the middle panel, the confirmed-catalogue triggers present in the CRC4 semblance stream, accumulated over all passes of the workflow, are added in black; these later discoveries fall in the seed region and immediately around it, consistent with the premise of the retrieval search (Section 2.3.3) that the triggers most worth reviewing are those nearest the confirmed events. The projection is illustrative rather than a proof: the gate itself works in the full four-feature space, where k-means groups the triggers and the clusters containing the seed events are retained, while the embedding is built only for display, after the gate is fixed, and never enters the scaling, screening, gating, or ranking (in production, projections of this kind were a final visual check among already-qualifying configurations, not the selection criterion). Its quantitative counterpart is the leave-one-out validation of Section 2.3.3, which tests the same co-location numerically on every pass. One thing the figure does not show is completeness: because the search retrieves candidates by their nearness to the seeds, the confirmed catalogue can only fill the part of the space that was searched, so the absence of black points far from the seed region reflects where review was directed under the project’s time limits, not a demonstrated absence of events there. The right panel zooms into this labelled region and re-colours the reviewed triggers by class: the triggers added during review spread across the same four categories as the seed events—dominated, as expected, by the abundant ocean-generated and surface-related classes—rather than concentrating in any single class, consistent with a retrieval search that returns the same mixture of coherent-wavefield types that seeded it.

### 3.3. Detector-Family Contributions

Table 6 breaks the final catalogue down by the well–detector stream that produced each participating trigger. The retained catalogue is dominated by the semblance family: 1156 of the 1325 catalogue triggers (87%) originate from the two semblance streams, and the dominance is most pronounced for the ocean-generated and regional earthquake classes—99% and 89% of triggers, respectively. STA/LTA contributes most where local amplitude contrast is strong, retaining a substantial share of induced-event triggers (7 of 16) and of strong surface triggers on CRC4.

This distribution supports the detector-design argument of Section 1 and Section 2.2: amplitude triggering and spatial-coherence detection view the same DAS record from different perspectives, and the classes that motivated DAS-specific detection—weak but spatially organised arrivals—are retained almost entirely through semblance. At the same time, the raw-trigger volumes of Table 4 show that detector choice alone cannot solve the screening problem: the semblance streams still produced 3.6 million raw triggers, and the value of each detector family was realised only after the trigger population was made searchable by the feature representation and the sparse-label workflow.

**Figure 6:**
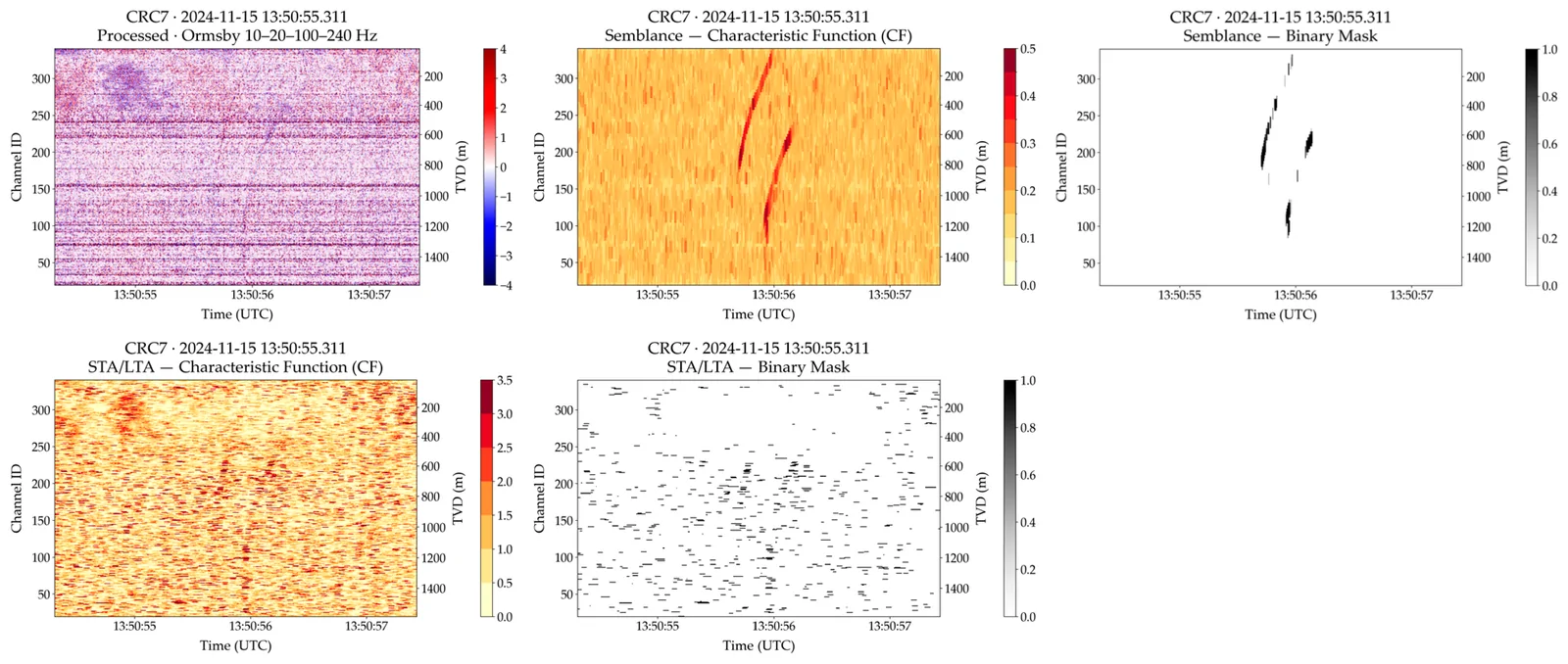
The two detector families applied to the same CRC7 recording of induced event 1 (15 November 2024). From left to right: the band-passed data panel; the semblance characteristic function and its binary mask, which isolate the two coherent arrivals of the event; and the STA/LTA characteristic function and its binary mask, which are dominated by dense single-channel transients that leave the event indistinguishable from the background.

Aggregate counts, however, conceal stream-specific failure modes that only the inspection of the detector outputs makes explicit. Figure 6 contrasts the two families on the CRC7 recording of induced event 1 (Section 3.5). On this stream the STA/LTA characteristic function is dominated by dense, randomly scattered single-channel transients; the resulting detection mask is an unstructured speckle field with no spatial organisation, and the event is indistinguishable from the background. The semblance characteristic function, computed from the same preprocessed data, isolates the two coherent arrivals of the event cleanly. This impulsive ringing—plausibly instrumental in origin, although its source was not investigated in this study—is what inflated the CRC7 STA/LTA stream to 10.2 million raw triggers (Table 4) and motivated setting that stream aside (Section 3.1). Identifying the source would have required instrument-level diagnostics and analysis of the raw, undecimated interrogator output, beyond the decimated archive processed here. Because semblance detection on the same recording remained fully usable, the investigation was deferred as its expected benefit did not justify the cost within the project window. The limitation is specific to the detector-noise pairing rather than to the well or the instrument: the same recording supports clean semblance detection.

**Figure 7:**
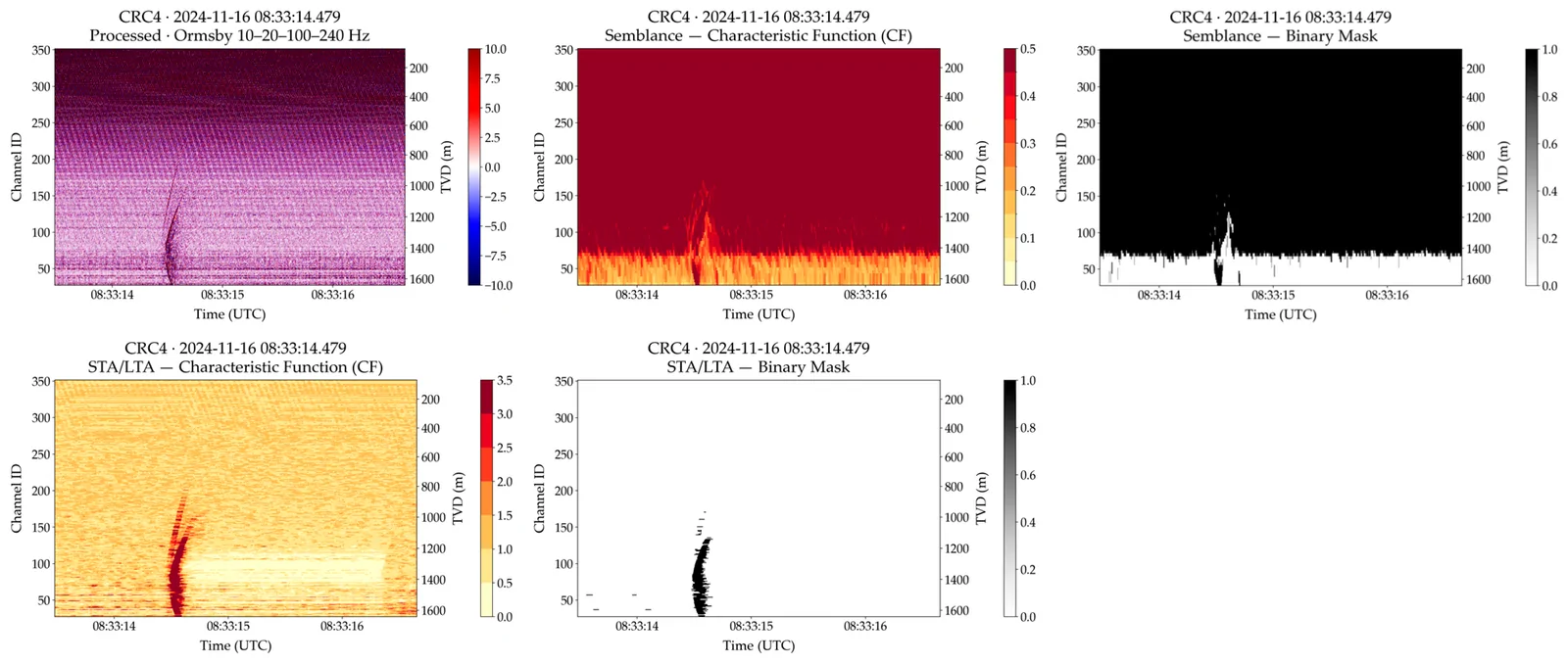
Induced event 3 (16 November 2024) recorded on CRC4 during operation of the surface orbital vibrators. The coherent orbital wavefield saturates the semblance characteristic function across most of the aperture and fuses its binary mask into a quasi-continuous orbital-pattern detection in which the event is not separable. STA/LTA absorbs the sustained orbital energy into its long-term average and isolates the impulsive event as a compact mask.

The complementary failure mode appears on CRC4 while the surface orbital vibrators (SOVs) installed at the site are operating. Figure 7 shows induced event 3 (Section 3.5) arriving during SOV operation. The orbital wavefield is itself spatially coherent, so the semblance characteristic function saturates across most of the aperture, and the binary mask fuses into a single quasi-continuous detection; the event does not emerge as a separable semblance trigger, and the fused detection that does form is—correctly, at trigger level—handled as orbital-pattern noise by the supervised gates of Section 2.3.3. STA/LTA, in contrast, normalises each channel by its own sustained background: the steady orbital energy is absorbed into the long-term average, and the impulsive event stands out above it as a compact, well-formed mask, so the event enters the catalogue through its STA/LTA detection. A geometry-guided detector that exploits predicted travel-time curves—such as the in-house screening tool of Section 2.4, or a template-based formulation like the 2D template-matching method [40]—could separate the event from the orbital background and would be a natural complementary detector here. The production semblance detector, by contrast, is deliberately a general-purpose local slant-stack over short, locally linear moveout segments: stacking over many short segments lets it approximate arrivals of arbitrary curvature and apparent velocity so that it generalises across coherent wavefields without assuming a cable geometry or a velocity model, at the cost of being unable to prefer one coherent wavefield over another. The two formulations are therefore complementary rather than competing, and no single detector family is privileged for routine use.

Taken together, Table 4 and Table 6 and Figure 6 and Figure 7 argue against selecting a single best well–detector combination. Each stream has noise regimes in which it is effectively blind and regimes in which it is uniquely sensitive, and the operative regime can change within hours as site operations and instrument behaviour change. Maintaining redundant streams is what protects recall, and the infrastructure decisions of Section 2.2 are what make it practical to do so: a deterministic front end that can re-traverse a multi-terabyte archive reproducibly, feature extraction that makes every stream searchable at the same fixed cost, and a triage layer that absorbs the order-of-magnitude trigger inflation that redundancy brings. In this design, adding a well, a detector family, or a new acquisition configuration enlarges the compute budget and the ranked review set; the analyst’s review effort still grows with the volume of retained data, but the triage layer is what keeps that growth far below the growth in raw trigger counts.

### 3.4. Per-Well Detectability

**Figure 8:**
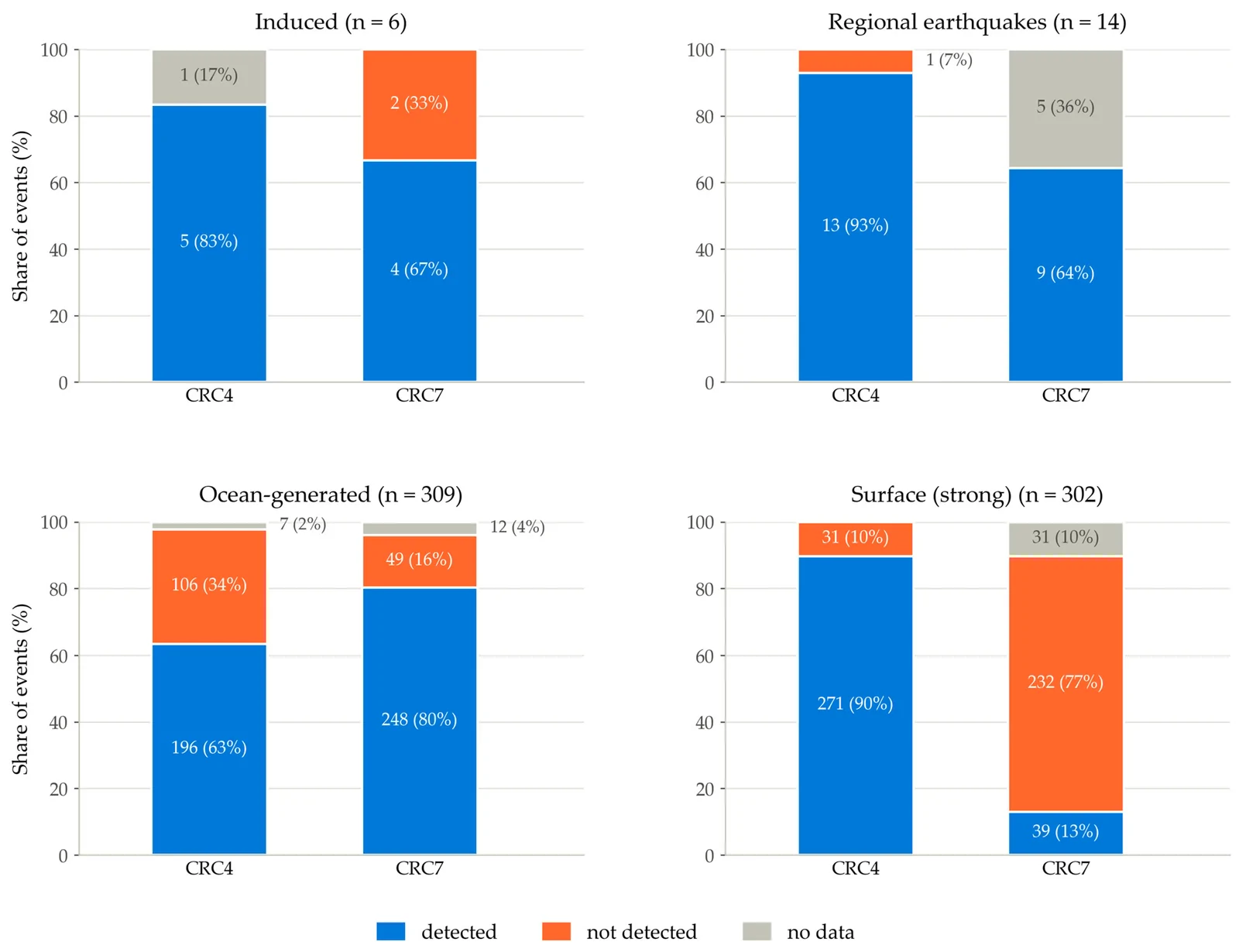
Per-well detectability of the 631 catalogue events by class, as a visual companion to Table 7. For each event class the two stacked bars give the share of CRC4 and CRC7 events in each status—detected, not detected (data available but no retained trigger), and no data (the well was not recording)—on a common percentage scale, with absolute event counts annotated on the bars. The contrast between wells is most pronounced for the strong surface-related class (retained mainly on CRC4) and the ocean-generated class (detected more often on CRC7).

Table 7, visualised in Figure 8, reports the per-well status of the 631 catalogue events, distinguishing detected (a retained trigger on that well), not detected (data available, but no retained trigger), and no data (the well was not recording over the relevant interval). Detectability differs systematically between wells and classes: strong surface-related events are retained predominantly on CRC4 (271 of 302), whereas ocean-generated events are detected more often on CRC7 (248 of 309). Induced events and regional earthquakes are largely visible on both wells wherever data exist. These per-well contrasts plausibly reflect the differing trajectory and deviation of the two wells, and hence the direction-dependent sensitivity of axial DAS strain measurement to wavefields arriving from different azimuths, although a quantitative directivity analysis was beyond the scope of this study.

Two operational points follow. First, the no-data category is part of the catalogue rather than a footnote: it separates true non-detections from coverage gaps, which is the distinction a monitoring programme needs when arguing completeness. Second, multi-well redundancy contributes directly to catalogue content—one of the six induced-event candidates falls within an interval with no usable CRC4 data and is represented by CRC7 alone (Section 3.5), while two further induced events (16 November 2024 at 05:32 and 28 November 2024) were recorded on CRC4 but not detected on CRC7 (Table 8). Redundancy therefore operates in both directions: neither well alone would have captured the full set of induced events.

### 3.5. Induced-Event Candidates and Comparison with Independent Template Matching

**Figure 9:**
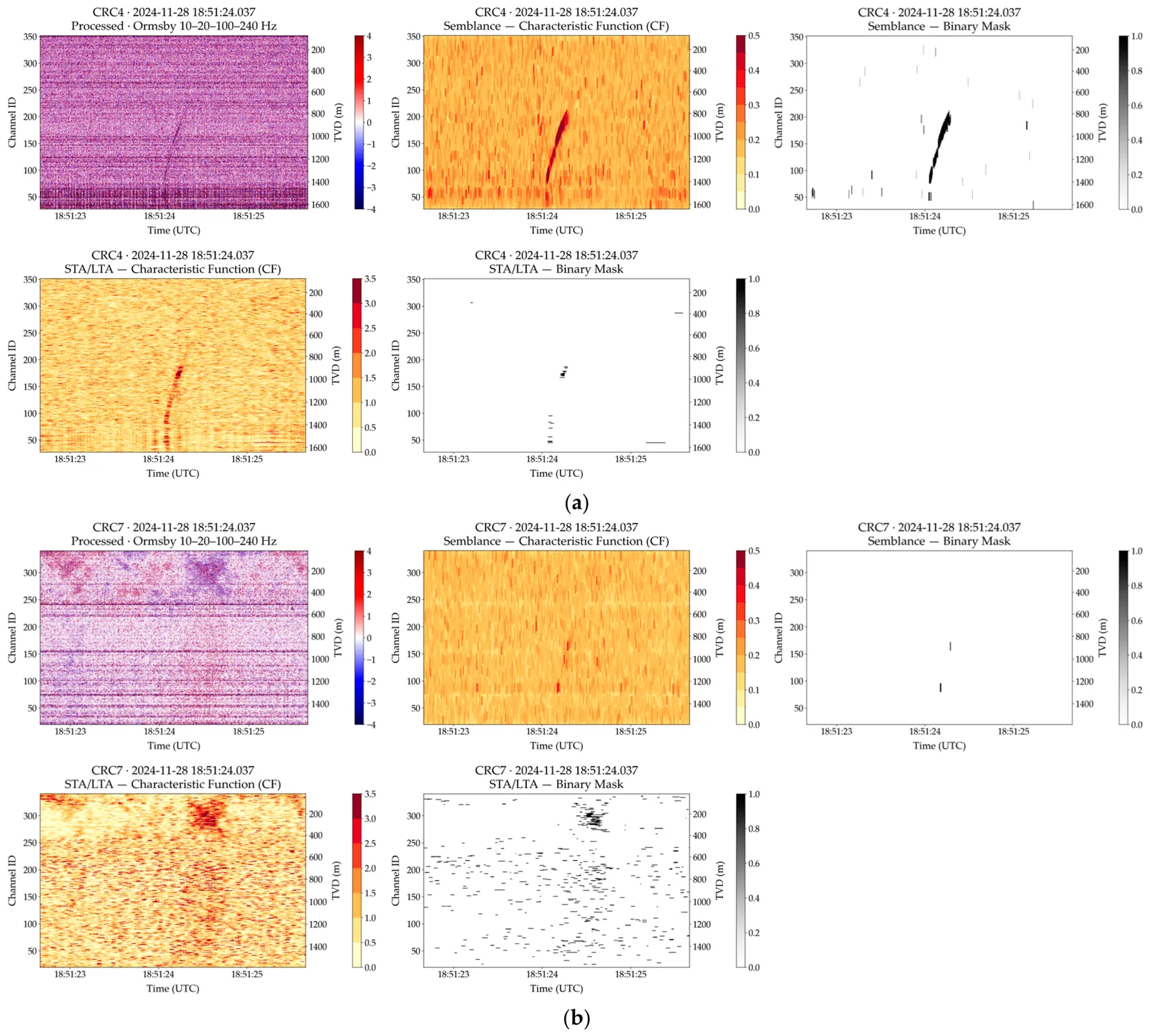
(**a**) Added induced-event candidate of 28 November 2024 recorded on CRC4, where the arrival is clearly expressed on both detector families; (**b**) the same 28 November 2024 candidate on CRC7, where it is not detected by either detector family, indicating a genuine limitation of coverage at this well rather than a detector-sensitivity artefact.

**Figure 10:**
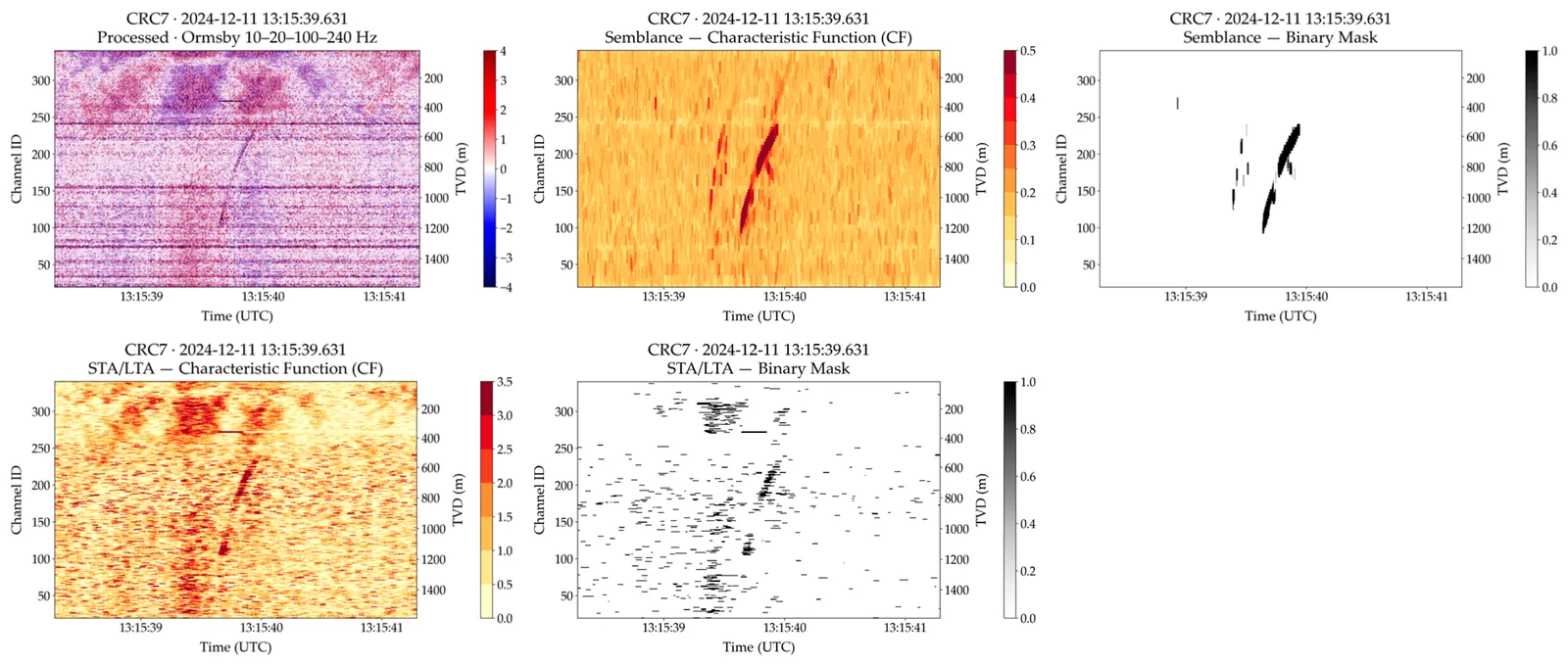
Added induced-event candidate of 11 December 2024 recorded on CRC7, where it is expressed predominantly on the semblance detector; CRC4 data were unavailable for this interval (Table 8).

The induced-event class is the smallest in the catalogue and the most consequential for storage monitoring. Table 8 lists the six induced-event candidates with their origin times and per-well status. All six occurred during the CO_2_ injection period (10 November 2024 to 21 January 2025), concentrated in the first weeks of injection. Four are the seed events of Section 2.4; the remaining two—on 28 November 2024 and 11 December 2024—were returned by the sparse-label workflow and confirmed during analyst review. Figure 9 and Figure 10 show the two added candidates with their full diagnostic context. For the 28 November 2024 event, both wells are shown—Figure 9a (CRC4) and Figure 9b (CRC7)—to make the cross-well comparison explicit: the event is clearly expressed on CRC4 but absent on CRC7; the independent CRC4-only template-matching analysis likewise did not flag this candidate (Table 8). This indicates a genuine limitation of CRC7 coverage for this event rather than an artefact of either detector.

The comparison with the independent template-matching analysis is informative in both directions. Template matching, applied to the raw (undecimated) CRC4 recordings with iteratively added templates, detected four induced events within the analysis window of this study; all four were present in the seed catalogue and retained by the workflow. The two candidates added by the sparse-label workflow arose through two different mechanisms and should not be grouped as a single template-matching miss. The 28 November candidate lies inside the template-matching analysis period on CRC4, so it is a like-for-like case: it was surfaced by feature-space retrieval and confirmed in analyst review, but was not detected under the template set and thresholds used in the independent CRC4 2D-TM analysis. The 11 December candidate, by contrast, occurred when CRC4 data were unavailable and was detected on CRC7, outside the CRC4-only scope of the template-matching analysis, so it reflects multi-well coverage rather than a template-matching miss.

This comparison should therefore be read as complementary rather than competitive. The sparse-label workflow first preserved every template-matching detection and then added two candidates through the two distinct mechanisms described above—feature-space retrieval within the template-matching coverage, and multi-well coverage beyond it. The result is neither a proof of catalogue completeness nor a general ranking of methods, but it shows that retrieval-based triage can add operational value around a highly sensitive template-matching analysis in sparse-label, multi-well DAS monitoring.

## 4. Discussion

### 4.1. Passive DAS Monitoring as an Operational Problem

Passive seismic monitoring with DAS is, in practice, an operational problem before it is a detection problem. A monitoring programme has a finite window in which to convert a continuous multi-well recording into a defensible event catalogue, and the same dense spatial sampling that makes a single fibre attractive as a seismic array is also what makes that conversion hard: the CRC4 and CRC7 recordings analysed here amounted to roughly 23 TB of decimated SEGY and yielded 14.14 million raw detector triggers over 120 days (Table 1 and Table 4). Exhaustive frame-by-frame review of a population of this size is neither feasible nor a sensible use of analyst time, yet the events that matter for storage monitoring are sparse within it. The problem the workflow addresses is therefore not how to label an event in isolation, but how to spend a limited review budget so that as many genuine coherent arrivals as possible are surfaced while the result remains reproducible and auditable.

Viewed this way, the contribution of the Argus workflow is a triage layer that operates as two successive reductions. The deterministic front end (Section 2.2) compresses the 23 TB archive—itself already a decimated, volume-reduced form of the raw interrogator output—into a 7.4 GB feature database of comparable trigger objects, after which the terabyte-scale data are revisited only to render quality-control panels for the few candidates that reach review. The sparse-label machine-learning layer (Section 2.3) then applies a second reduction, retrieving from those millions of triggers the few thousand most similar to confirmed events and presenting them as a ranked review set sized to a one-to-two-day inspection budget. This two-stage funnel is what allows the volume of DAS data to stop being a bottleneck to its passive-monitoring use: the analyst effort scales with the volume of retained, plausibly informative data rather than with the raw trigger count, even as wells, detector families, or acquisition configurations are added. The operational value of the workflow is consequently better measured by this reduction in reviewable volume, achieved without sacrificing provenance, than by any single detection-accuracy figure.

### 4.2. Relationship to Existing Detection and Learning Approaches

Machine-learning methods for DAS event detection have advanced rapidly, and it is useful to state where a retrieval-first, sparse-label workflow sits among them. Supervised deep-learning detectors have been applied successfully to microseismic and passive DAS data, including in settings closely related to the present one [23,24]. Their effectiveness, however, presupposes a training population that is either large enough to represent the target class or validated against substantial independent field evidence, together with a negative class that can be defined well enough to estimate false-negative behaviour. Early-stage passive monitoring of a small CO_2_ injection does not yet meet these conditions: confirmed induced events are few, the negative class is heterogeneous and initially unlabelled, and the absence of a detection by a trained classifier cannot be read as evidence that no coherent event occurred. The workflow is therefore best understood not as an alternative to supervised learning, but as the stage that can precede it. It operates while labels are still too few to train a classifier—and, more importantly, to validate one—and it produces the analyst-confirmed events and named noise families that such a classifier would later need. Supervision then plays a deliberately narrow role: the supervised gates (and, once enough labels accumulate, a gradient-boosted classifier; Section 2.3.3) only reject specific, already-recognised noise families, and only after retrieval and coarse gating have concentrated the candidate set—they are never imposed from the outset as a global classifier. Accumulating negative labels sharpens these local gates but does not close the open-set negative-class problem: the unlabelled population remains heterogeneous and may still contain noise families and events that no gate has yet seen.

A similar distinction separates the workflow from waveform-correlation and template-matching approaches, which have long been among the most sensitive detectors for repeated or co-located seismicity [27,28,29] and which provided part of the seed catalogue used here (Section 2.4). Template matching is, by design, tuned to a specific question—whether a new signal is a waveform-level repeat of a known one—and is therefore most sensitive to events that share source location, mechanism, path, and recording response. Retrieval in the interpretable feature space of Section 2.2 addresses a different question, ranking triggers by similarity in abstracted geometric and physical character rather than by waveform shape so that events of the same kind can be surfaced without being near-repeats of any single master waveform. The catalogue expansion as a whole is consistent with this broader retrieval behaviour, and the workflow first preserved every template-matching detection; however, the induced-event comparison itself (Section 3.5, Table 8) is too small and not fully like-for-like to serve as a performance benchmark. The relationship is thus complementary rather than competitive: property-space retrieval complements a highly sensitive but waveform-specific method by surfacing candidates that are close in feature space without being waveform-level repeats, and the comparison should be read as evidence of added operational coverage rather than as a ranking of detectors.

In machine-learning terms, the early-monitoring regime is a positive–unlabelled problem [31]: reliable positive examples exist, negatives do not, and both supervised training and supervised evaluation remain ill-posed until negative labels accumulate. Ranking unlabelled triggers by their similarity to confirmed positives is a natural response to this structure, and the analyst-labelling loop that returns new positives and negatives places the workflow within human-in-the-loop learning [33]. It departs deliberately from classical active learning [32] in how it selects what to review: batches are chosen by similarity to confirmed events rather than by model uncertainty. Under the cost structure of this task—where reviewing additional noise is cheap but discarding a scarce induced-event example is not—concentrating review where genuine events are most likely is more defensible than sampling the decision boundary, even though the latter would in principle be more informative for a future classifier. Finally, the decision to build the representation from interpretable attributes rather than to learn one end-to-end follows the argument of Rudin [30] that high-stakes, review-critical decisions are better served by transparent models; Section 4.3 returns to why this matters specifically for storage assurance.

### 4.3. Implications for CO_2_ Storage Monitoring

For geological CO_2_ storage, the events this workflow surfaces are relevant on two levels. Induced seismicity is at once a risk-management concern—through the possibility of fault reactivation or the creation of leakage pathways—and a potential diagnostic of pressure diffusion and geomechanical response to injection [19,20]. The other coherent-wavefield classes retained by the catalogue (regional earthquakes, ocean-generated and strong surface-related events) are not injection signals, but their systematic identification is what allows induced events to be distinguished positively rather than by exclusion, and what gives the per-well detectability accounting of Section 3.4 its meaning.

The less obvious implication concerns what a storage-monitoring programme actually needs from a detection layer. Containment assurance and storage conformance are evidentiary tasks carried out over the long operating life of a store and subject to external scrutiny [1,2,3,5]: the requirement is not only that events be detected, but that the catalogue be reproducible, conservatively interpreted, traceable to its source data, and open to reprocessing as understanding and labels improve. These are precisely the properties the workflow was built to preserve—every accepted event remains linked to its trigger identifier, detector stream, feature row, processing configuration, and source data (Section 2.3.4), and the deterministic front end can re-traverse the archive reproducibly as objectives evolve. In this setting an auditable candidate-ranking workflow whose acceptance step is a recorded human decision is arguably more defensible than an opaque classifier of comparable or even superior nominal accuracy, because the quantity that matters most for assurance—false-negative behaviour under previously unseen noise and acquisition conditions—is exactly what cannot be validated for such a classifier while labels remain sparse. The conservative reporting adopted here, in which scarce-class counts are presented as a reviewed lower bound rather than a census (Section 3.2 and Section 4.6), reflects the same posture: because neither completeness nor false-negative behaviour can be independently validated under sparse labels, conservative interpretation is preferable to unsupported inflation of the catalogue—a consideration that carries particular weight where a claim of induced seismicity has consequences beyond the scientific record. By making continuous review tractable, the workflow also makes passive DAS monitoring a practical, repeatable complement to the active time-lapse imaging that the same installed fibre supports [7,10,11,12], rather than a modality whose data volume confines it to occasional, selective analysis.

### 4.4. Feature-Space Generalisation Under Sparse Labels

The workflow rests on a hypothesis that the results test rather than assume: that induced, regional earthquake, ocean-generated, and strong surface-related events, despite very different sources and scales, can be pooled into a single labelled family of coherent wavefields because they share the abstract attributes that separate organised arrivals from clutter—coherent moveout, mask continuity, and structured spectral content (Section 2.4). Pooling is what makes the search tractable at all, lifting the effective seed support from four induced examples to thirty-nine confirmed events so that the scarcest and most consequential class can be sought alongside the others rather than alone. Because the search is directed by these seeds, assembling them is the genuine entry cost of the approach, and the fact that they were produced by methods independent of Argus (Section 2.4) matters for rigour: the workflow uses externally confirmed events as queries rather than rediscovering its own training examples, which limits the circularity to which retrieval- and template-based methods are otherwise exposed.

The evidence is consistent with this hypothesis without proving it, and the distinction governs how the catalogue should be read. The leave-one-out validation of Section 2.3.3 and the projection of Figure 5 both show the confirmed classes concentrating in a shared region into which later discoveries fall—the empirical basis for the broader observation that retrieval by abstracted properties can generalise across event morphologies in a way that waveform correlation, by construction, cannot. This is observed rather than demonstrated: the search is seeded by known events and is therefore disposed to find their neighbours, and the projection is illustrative rather than the space in which the gates operate (Section 3.2). The strongly uneven expansion across classes (Table 5) is best interpreted not as the method amplifying some classes over others but as a reflection of how often each wavefield actually recurs over a 120-day window—frequently for ocean-generated and surface arrivals, rarely for induced events and regional earthquakes—though this too is an interpretation the data support rather than establish.

### 4.5. Detector Redundancy and the Role of Scalable Infrastructure

A recurring theme of the results is that detector choice and workflow architecture cannot be considered separately. The two detector families viewed the same recordings differently and failed differently: semblance supplied the large majority of retained catalogue triggers and almost all of the spatially coherent regional and ocean-generated arrivals, while STA/LTA remained the only stream to retain certain compact induced events recorded during strong coherent background activity (Section 3.3, Table 6). Crucially, each family was effectively blind in some noise regimes and uniquely sensitive in others, and the operative regime could change within hours as site operations and instrument behaviour changed. The methodological lesson is that no single well–detector combination should be privileged for routine passive monitoring; recall is protected by maintaining redundant streams and by allowing unproductive ones to be set aside after quality control, rather than by searching for a single optimal detector.

Redundancy of this kind is affordable only because of the infrastructure that surrounds it, and this is where the architecture becomes a scientific rather than merely an engineering contribution. Adding wells, detector families, or preprocessing configurations multiplies the raw trigger count—the semblance streams alone produced 3.6 million triggers (Table 4)—and such inflation is tolerable only because the deterministic front end can re-traverse the multi-terabyte archive reproducibly and the sparse-label layer can absorb the additional triggers at a fixed per-trigger cost. The same separation carries a second benefit: once the single expensive contact with the raw SEGY archive has produced the trigger-level feature database, downstream experiments on feature screening, retrieval strategy, supervised noise gates, and detector combinations can be repeated without re-reading terabytes of waveform data, while every result remains linked back to the original record. Catalogue expansion under sparse labels is, in this light, a multi-method undertaking—template matching, travel-time-guided screening, detector triggering, and feature-space retrieval each expose a different part of the event population (Section 3.5)—and it is the front-end-to-feature-database architecture that makes combining and comparing those methods practical at field scale.

### 4.6. Catalogue Validity and Limitations

Two validity questions follow from the design: whether the added events are real, and whether important events were missed. On the first question, no event entered the catalogue on the strength of a model score alone. The machine-learning layer controlled only the order and volume of review: each of the 592 added events was individually confirmed by an analyst on the multi-panel quality-control plots, and every acceptance is recorded against a persistent trigger identifier (Section 2.3.4). The residual false-positive risk is therefore analyst misclassification, discussed as the fourth limitation below, rather than unreviewed model output. For the scarce classes, the added candidates were examined against all available evidence from both wells and both detector families. For the induced class, where a false acceptance carries the greatest consequence, the two added candidates were additionally examined against the independent template-matching analysis, with non-detections and data gaps reported explicitly (Section 3.5, Table 8).

On the second question, completeness cannot be established under sparse labels, and the catalogue is reported as a reviewed lower bound. What can be verified is behaviour on the independently confirmed references that exist: the seed triggers present in each processed stream were protected by the recall floors, premium-label protection, and margin-safety constraints of Section 2.3.3; all 39 seed events remained represented in the final catalogue; and all four induced events detected by the independent template-matching analysis were preserved, with two further candidates added (Section 3.5). These checks verify retention of the available independently confirmed positive references; they do not bound false-negative behaviour beyond those references, for which the first limitation below applies.

Several limitations follow directly from the problem setting and the design and are stated here so that the catalogue is read with appropriate caution.

First, completeness cannot be established, and this is a property of the task rather than a shortfall of the implementation. A retrieval search seeded by confirmed events is inherently directed towards the neighbourhood of those events, so the absence of confirmed events far from the seed region (Section 3.2) reflects where review was directed under the project’s time constraints, not a demonstrated absence of events there. Completeness could only be proven by the exhaustive manual review that the workflow exists to avoid; the scarce-class counts should therefore be treated as a reviewed lower bound, and the workflow makes no claim to have recovered every coherent event in the record. The supervised noise gates do not change this: the labelled noise set is incomplete and opportunistically accumulated, the gates were trained only for recognised recurrent artefact families and should not be interpreted as exhaustive noise modelling, and neither completeness nor false-negative behaviour can be guaranteed over the full unlabelled trigger population.

Second, the results depend on the seed catalogue. The seeds used here were obtained independently of the workflow (Section 2.4), which is a strength for validation but also means that the regions of feature space the search explores were fixed by that initial selection. A different seed set could surface different parts of the population, and the pooled-family assumption of Section 4.4 remains a working hypothesis whose generality beyond this dataset is untested. This directional bias is intrinsic to retrieval- and template-based methods rather than peculiar to this implementation and is mitigated—though not removed—by diversifying the seed catalogue across independent sources (Section 4.7).

Third, the influence of detector configuration on the catalogue was not characterised. The characteristic-function thresholds of the STA/LTA and semblance detectors were set permissively so that the detectors recovered all available seed events (Section 2.2), but the sensitivity of the resulting trigger population—and hence of the expanded catalogue—to these thresholds was not studied. Whether sweeping them would surface additional events or merely enlarge the noise burden is an open question.

Fourth, the workflow is human-in-the-loop by design, and final event identity and acceptance rest on analyst judgement exercised against the quality-control panels (Section 2.3.4). This introduces a degree of subjectivity that the automation does not remove: visual classification of recurring time–channel patterns can vary between analysts, and the catalogue reflects the consistent judgement of the reviewers involved rather than an inter-rater-validated standard. The design mitigates but does not eliminate this dependence—every decision is recorded against a persistent trigger identifier and can be revisited or re-adjudicated as the catalogue matures—but the labels that drive the search are themselves human products and carry that variability into the screening and ranking.

Fifth, this study covers a single site and a single pair of wells. Because DAS records strain along the fibre and its response depends on cable geometry, coupling, gauge length, interrogator characteristics, and local velocity structure [14,15], neither the feature representation nor the seed events can be assumed to transfer unchanged to other deployments; cross-cable transfer is therefore raised as future work in Section 4.7 rather than claimed here.

Sixth, the feature representation is operationally useful rather than optimal or universal. The 67 attributes were hand-designed to be interpretable and computed once as a reusable embedding (Section 2.2); the per-task screening selects among them empirically rather than establishing that any subspace is the best available, and the transformation and scaling are re-fitted as the labelled population grows (Section 2.3.1), introducing a dependence of the feature geometry on the evolving catalogue that was not separately ablated. The space should be understood as an interpretable abstraction of DAS image-like wavefield character, not as a general representation of seismicity.

Finally, one stream was set aside on pragmatic grounds. The CRC7 STA/LTA stream, dominated by dense single-channel impulsive transients of plausibly instrumental origin, contributed more than 70% of all raw triggers and was excluded from ML-assisted expansion after quality control (Section 3.1 and Section 3.3). The decision is well supported by the structure of that stream, but it is a choice, and the source of the impulsive ringing was not investigated in this study.

### 4.7. Future Directions

A first extension is cross-deployment transfer of the search. The feature representation is dimensionless after transformation and scaling, so events confirmed on one cable could in principle seed a search on another. A dimensionless representation is not transferable, however: cable geometry, coupling, gauge length, interrogator response, and local velocity structure all shape the recorded signature, so any transfer would have to be established empirically, after site-specific re-normalisation and validation. If it holds, the cost of opening a new monitoring deployment, currently dominated by assembling a site-specific seed catalogue, would fall substantially.

A second extension concerns detection. The general-purpose local slant-stack semblance detector used here generalises across coherent wavefields without assuming a geometry, but for exactly that reason it cannot separate an induced event from the spatially coherent wavefield of the surface orbital vibrators (Figure 7). A complementary geometry-guided detector that exploits predicted travel-time curves—as the in-house screening tool does, and as template-based methods such as [40] do—would address this case and could be added as a further detector family within the same deterministic front end, computed on data already held in memory rather than by re-traversing the archive.

Three further refinements would tighten the workflow itself. The sensitivity of the catalogue to detector thresholds (Section 4.6) deserves a dedicated study to establish whether threshold sweeps recover additional events at an acceptable noise cost. Seed building could combine travel-time-guided screening, template matching, public earthquake-catalogue windows, and targeted manual review so that the search is not anchored to a single source of confirmed events. Analyst review could be formalised through measured inter-analyst agreement, class-specific acceptance criteria, and versioned review guidelines, making the human contribution as auditable as the automated stages.

With site-specific experience and cross-cable evidence, the same infrastructure could move towards near-real-time operation alongside the interrogator; autonomous detection would require validation across geometries and noise conditions that a single-site study cannot provide. The triage problem itself is not specific to CO_2_ storage: comparable data volumes and label scarcity arise in geothermal and reservoir surveillance, volcanic monitoring, and long-duration infrastructure deployments, and the same structure may apply there, provided the feature representation and seed events are revalidated for each new setting.

## 5. Conclusions

This study presented Argus, a sparse-label machine-learning workflow for expanding passive DAS event catalogues under the label-scarce, high-volume conditions typical of early-stage CO_2_ storage monitoring, and applied it to the CO2CRC Otway Stage 4 dataset. The workflow separates the task into a deterministic front end, which converts a multi-terabyte DAS archive into reproducible trigger objects with interpretable features, and a retrieval-first machine-learning layer, which ranks those triggers by similarity to a small seed catalogue of independently confirmed events and routes the most promising to analyst review. Rather than imposing supervised classification from the outset, the workflow combines retrieval-first triage with optional, local supervised rejection of labelled recurrent artefact families. These gates are added only as analyst review supplies the labels that make them well-posed, and the gates are never used as a global classifier over the full unlabelled trigger population.

On the Otway data, the workflow reduced 14.14 million raw triggers to review sets of a few thousand candidates and expanded a 39-event seed catalogue into 631 analyst-reviewed events, demonstrating complementarity with an independent template-matching analysis: two additional induced-event candidates were recovered, one within the CRC4 template-matching coverage and one on CRC7 during a CRC4 data gap. The expansion is dominated by the recurrent ocean-generated and surface-related classes; the induced-event class itself grew only from four to six candidates, and its counts are reported as a reviewed lower bound rather than a complete census. The detector and per-well analyses showed that no single well–detector combination is sufficient, and that redundant streams—made affordable by the scalable front end—are what protect recall across changing noise regimes.

The central contribution is a reproducible mechanism for converting continuous DAS recordings into an auditable event inventory. Every accepted event remains traceable to its trigger identifier, detector stream, feature row, and processing configuration, and the deterministic front end can re-traverse the archive as labels and objectives evolve. For CO_2_ storage assurance, where catalogues face external scrutiny over long operating periods, these properties matter as much as raw sensitivity, and the labelled events and named noise families accumulated by the loop provide the basis on which future site-specific supervised models can be trained and validated.

## Figures and Tables

**Table 1:** DAS dataset analysed in this study. The analysis used representative downhole intervals from CRC4 and CRC7 over a common 120-day window spanning baseline, injection, and post-injection monitoring.

Parameter	CRC4	CRC7
Monitoring period analysed	1 November 2024 to 1 March 2025	
Stage 4 injection interval	10 November 2024 to 21 January 2025	
Channels analysed	322	319
Nominal channel spacing	≈5.1 m	
Downhole sensing aperture	≈1.6 km	
Sample interval	2 ms (500 Hz)	
Workflow input archive	Decimated SEGY	
Scanned archive size	11.9 TB	11.2 TB

**Table 2:** The 67 interpretable features computed per trigger, grouped into 13 semantic families, with the measured trigger property and an illustrative interpretation of the contrast each axis can express on the two-dimensional time–channel DAS panel.

Feature Family (Prefix)	*n*	Measured Trigger Property	Illustrative Interpretation
Geometry (geo)	4	First-order footprint geometry: trigger duration, channel (depth) span, centre depth (the midpoint between the trigger’s shallowest and deepest channels), and time–depth aspect ratio.	Footprint size and shape—compact, short-duration patterns versus long-duration or vertically extensive footprints, and the time-versus-depth elongation between them.
Mask morphology (mask)	14	Detector-mask occupancy, shape and activation pattern, e.g., body area, fill ratio, multi-channel coincidence, active-channel count, spatial continuity, largest channel gap, activation timing, and convex-hull solidity.	Mask quality and organisation—a coherent, well-filled, single body with channels activating together versus a ragged, gappy, sparsely filled mask with uneven activation.
Waveform statistics (wave)	8	Amplitude distribution over the full trigger ROI (independent of the mask): RMS, peak, crest factor, kurtosis, skewness, and channel- and time-domain RMS variability.	Amplitude texture of the panel—impulsive, concentrated energy (high crest factor/kurtosis) versus diffuse, sustained or evenly distributed energy.
Amplitude and SNR (amp)	8	Mask-aware signal strength relative to the local pre-trigger background: RMS and peak signal-to-noise ratio, masked-signal and in-ROI noise RMS, peak amplitude, crest factor, energy concentration, and inter-channel energy variability.	Signal strength on the panel—strong masked responses that stand clearly above the local background versus weak, near-threshold crossings; and energy concentrated on the masked body versus spread across the ROI.
Energy envelope (env)	5	Temporal energy-envelope shape: rise time, decay time, peak-time position, energy half-width, and temporal entropy.	Energy-envelope shape—sharp-onset, short-coda pulses versus slowly emerging, sustained or broadly modulated energy.
Wavefront shape (shape)	6	Leading- and trailing-edge geometry of the detector mask: front and tail slope, linearity (R^2^), and curvature.	Wavefront-edge geometry—near-linear (planar) mask edges versus curved or poorly organised leading and trailing edges.
Moveout (mov)	2	Apparent channel-to-channel propagation from adjacent-channel FFT cross-correlation: moveout fit quality (R^2^) and apparent slope/slowness (ms/m).	Apparent moveout—a consistent lag trend across channels versus weak or disordered moveout; slope sign and magnitude give propagation direction (up- or down-going) and apparent slowness.
Background (bg)	3	Pre-trigger background over the trigger’s active channels: background RMS, spectral centroid, and channel-to-channel variability.	Background context the candidate sits in—a quiet, spectrally low, uniformly coupled neighbourhood versus a noisy or spatially variable one.
Spectral (spec)	5	Frequency content of a representative active-channel trace: centroid, bandwidth, 85% roll-off, spectral flatness, and a validity flag.	Frequency-band character—narrow-band versus broadband, and high- versus low-frequency content; the validity flag marks reliable spectral estimates versus triggers too short to transform.
Coherence (coh)	3	Channel-to-channel waveform similarity and its decay with channel separation: neighbour-correlation mean and median, and correlation decay rate.	Spatial organisation—waveforms that repeat coherently from channel to channel versus single-channel or incoherent disturbances.
Fragmentation (frag)	2	Connected-component structure of the detector mask: number of components and fraction of the mask contained in the largest component.	Spatial integrity—a single connected body versus scattered, multi-fragment detections.
Quality (qual)	4	Boundary/truncation flags: whether the trigger touches the chunk edge in time or channel (possible truncation).	Provenance and QC context for potentially truncated candidates; excluded from the similarity and scaling subspaces in this study and handled separately.
Context (ctx)	3	Detector-stream temporal neighbourhood: gap to the previous trigger, gap to the next trigger, and local trigger density.	Temporal context—isolated arrivals versus bursts or recurrent, closely spaced activity.
**Total**	**67**		

**Table 3:** Machine-learning design choices and their role in sparse-label passive DAS monitoring.

Design Choice	Purpose	Why It Matters
Interpretable trigger features	Represent time–channel morphology numerically	Supports geophysical review and avoids opaque end-to-end decisions
Robust transform and scale	Condition features for distance-based learning	Prevents amplitude or duration features from dominating distances arbitrarily
Task-specific screening	Select discriminative subspaces for each ML task	Avoids reusing stale or irrelevant feature rankings
High-recall k-means gate	Remove obvious clutter while protecting scarce target labels	Matches early-monitoring conditions with incomplete field labels
Margin safety for premium labels	Reject gates that place important labels near non-target boundaries	Protects induced-event candidates and other high-value labels
Human-in-the-loop validation	Use ML for prioritisation, not autonomous catalogue declaration	Provides auditability needed for scientific and industrial use

**Table 4:** Raw triggers per well and detector stream over the 120-day analysis window.

Well	Detector	Raw Triggers
CRC4	STA/LTA	265,762
CRC4	Semblance	2,043,507
CRC7	STA/LTA	10,233,590
CRC7	Semblance	1,602,024
**Total**		**14,144,883**

**Table 5:** Catalogue expansion by event class, from the seed catalogue (Section 2.4) to the final analyst-reviewed catalogue.

Category	Seed Events	Final Events	Added	Expansion
Induced	4	6	+2	1.5×
Regional earthquakes	11	14	+3	1.3×
Ocean-generated	7	309	+302	44.1×
Surface (strong)	17	302	+285	17.8×
**Total**	**39**	**631**	**+592**	**16.2×**

**Table 6:** Triggers participating in the final catalogue, by event class and well–detector stream.

Category	Events	Triggers	CRC4 Semblance	CRC4 STA/LTA	CRC7 Semblance	CRC7 STA/LTA
Induced	6	16	5	4	4	3
Regional earthquakes	14	132	50	12	67	3
Ocean-generated	309	451	199	2	248	2
Surface (strong)	302	726	545	140	38	3

**Table 7:** Per-well detection status of the final catalogue events (detected/not detected/no data).

Category	CRC4 Detected	CRC4 Not Detected	CRC4 No Data	CRC7 Detected	CRC7 Not Detected	CRC7 No Data
Induced	5	0	1	4	2	0
Regional earthquakes	13	1	0	9	0	5
Ocean-generated	196	106	7	248	49	12
Surface (strong)	271	31	0	39	232	31
**Total**	**485**	**138**	**8**	**300**	**283**	**48**

**Table 8:** Induced-event candidates in the final catalogue. 2D-TM: independent 2D template-matching analysis of the raw CRC4 recordings (method of [40]), covering 1 October 2024 to 6 January 2025 with 9–15 December 2024 unavailable.

Event	Origin Time (UTC)	CRC4	CRC7	Provenance	2D-TM [40]
1	15 Nov 2024 13:50:55	detected	detected	seed	detected
2	16 Nov 2024 05:32:54	detected	not detected	seed	detected
3	16 Nov 2024 08:33:14	detected	detected	seed	detected
4	26 Nov 2024 10:38:59	detected	detected	seed	detected
5	28 Nov 2024 18:51:24	detected	not detected	ML-assisted review	not detected
6	11 Dec 2024 13:15:39	no data	detected	ML-assisted review	no data (CRC4)

## Data Availability

Restrictions apply to the availability of these data. Data were obtained from Stage 4 of the CO2CRC Otway project and are available from the authors with the permission of CO2CRC Limited.

## Appendix A. Trigger-Generation Parameters

This appendix records the deterministic front-end parameters used for the Otway Stage 4 production runs (Section 2.2). All values are fixed for the whole 120-day archive and are stored with every resulting trigger for reproducibility. The sample interval is 2 ms (500 Hz) throughout.

**Preprocessing.** Robust per-channel amplitude clipping based on the median and median absolute deviation (MAD), clipping samples beyond 50 robust standard deviations (50 × 1.4826 × MAD; about 0.01% of samples) to remove instrument spikes, followed by a zero-phase Ormsby band-pass filter with corner frequencies 10–20–100–240 Hz applied in overlapping FFT blocks of 8192 samples.

**STA/LTA detector.** Classic short-term-average/long-term-average ratio with a 50 ms short-term window (25 samples), an 1800 ms long-term window (900 samples), a trigger-on ratio of 3.5 and a trigger-off ratio of 2.0, with one long-term window of warm-up before detections are accepted.

**Semblance detector.** Neidell–Taner semblance computed in a 20 ms (10-sample) sliding time window over moving groups of 16 channels (about 80 m aperture) stepped 4 channels at a time (75% overlap), scanning 41 apparent-slowness values uniformly spanning ±1 × 10^−3^ s/m (apparent velocities |v| ≥ 1000 m/s); a trigger is declared where the semblance exceeds 0.35.

**Trigger assembly.** Detector masks are projected onto the time axis and grouped into trigger bodies against a causal rolling background (50 s window, 15th percentile), requiring at least 5 active channels and an excess of at least 3 channels (or 5% of the local background, whichever is larger) above that background, with runs closer than 50 ms merged.

**Geometric constraints.** Triggers smaller than a permissive physical floor were discarded before feature extraction: a minimum channel span of 20 channels (about 100 m) and a minimum spatial fill (body area/bounding-box area) of 0.02, together with a minimum duration of 30 ms (Section 2.2). These floors were set well below the smallest confirmed seed event so that weak but spatially coherent candidates were retained.

## Appendix B. Feature Definitions

Each trigger is described by a fixed 67-feature vector organised into 13 semantic families (Table 2). The features are computed once, from the waveform samples within the trigger footprint and a short pre-trigger background window, at extraction time. Table A1 defines every feature and how it is computed. Here dx is the channel spacing (about 5.1 m), dt the sample interval (2 ms), the region of interest (ROI) is the trigger’s time–channel bounding box, the mask (body) is the detector-defined active region within it, and active channels are those carrying at least one mask sample; amplitude statistics in the amp and bg families use active channels only.

**Table A1:** Definitions of the 67 per-trigger features, grouped by family.

Feature	Definition/Computation
**Geometry (geo)—4 features**	
geo.duration_ms	Trigger duration: (number of time samples in the ROI) × dt.
geo.channel_span_m	Spatial extent: (number of channels − 1) × dx.
geo.center_depth_m	Measured depth of the channel midway between the trigger’s shallowest and deepest active channels (from the survey geometry).
geo.aspect_ratio	duration_ms/max(channel_span_m, dx): time-versus-depth elongation.
**Mask morphology (mask)—14 features**	
mask.body_area	Number of active (masked) time–channel pixels in the detector body.
mask.fill_ratio	body_area divided by the ROI area (n_time × n_channels).
mask.coincidence_peak	Maximum number of channels active in any single time sample.
mask.coincidence_mean	Mean number of channels active per time sample.
mask.triggered_channels	Number of distinct channels carrying at least one mask sample.
mask.spatial_continuity	n_active/(index span of active channels), clipped to the 0–1 range; 1 = a contiguous channel block.
mask.max_channel_gap_m	Largest gap between consecutive active channels × dx.
mask.temporal_consistency	Mean over active channels of (samples active in that channel/ROI duration in samples).
mask.temporal_uniformity	1 − std/mean of the per-sample active-channel count (clipped to the 0–1 range); evenness of activity through time.
mask.coincidence_efficiency	coincidence_peak/triggered_channels (clipped to the 0–1 range); ≈1 = near-simultaneous (burst-like) activation, lower values indicate moveout.
mask.front_loaded_ratio	Fraction of body_area falling in the first third of the ROI duration.
mask.back_loaded_ratio	Fraction of body_area falling in the last third of the ROI duration.
mask.peak_time_ratio	Time of the peak active-channel count, normalised to the 0–1 range over the ROI.
mask.solidity	body_area divided by the area of its convex hull; 1 = a compact, tight footprint, low values = scattered.
**Waveform statistics (wave)—8 features**	
wave.roi_rms	RMS amplitude over all samples of the ROI (independent of the mask).
wave.roi_peak_abs	Maximum absolute amplitude in the ROI.
wave.roi_crest_factor	roi_peak_abs/roi_rms.
wave.roi_kurtosis	Excess kurtosis of the ROI amplitude distribution (impulsiveness).
wave.roi_skewness	Skewness of the ROI amplitude distribution.
wave.channel_rms_cv	Coefficient of variation (std/mean) of the per-channel RMS.
wave.time_rms_cv	Coefficient of variation of the per-time-sample RMS.
wave.abs_p95_over_median	95th percentile divided by the median of |amplitude| over the ROI.
**Amplitude and SNR (amp)—8 features**	
amp.snr_rms	rms_signal divided by the pre-trigger background RMS (active channels).
amp.snr_peak	Peak masked amplitude divided by the background RMS.
amp.peak_amplitude	Maximum absolute amplitude inside the mask.
amp.rms_signal	RMS amplitude of the samples inside the mask.
amp.rms_noise_roi	RMS amplitude of the samples inside the ROI but outside the mask.
amp.crest_factor	peak_amplitude/rms_signal for the masked body.
amp.energy_concentration	Masked energy as a fraction of total ROI energy.
amp.channel_energy_cv	Coefficient of variation of per-channel masked energy over active channels.
**Energy envelope (env)—5 features**	
env.rise_time_ms	Time from ROI start to the peak of the masked energy envelope.
env.decay_time_ms	Time from the envelope peak to the ROI end.
env.peak_time_ratio	Envelope-peak time normalised to the 0–1 range over the ROI.
env.energy_half_width_ms	Total time for which the envelope exceeds half its peak value.
env.temporal_entropy	Shannon entropy of the normalised energy envelope (impulsive vs. sustained).
**Wavefront shape (shape)—6 features**	
shape.front_slope_ms_per_ch	Slope of a linear fit of first-active-sample time versus channel (onset moveout), in ms per channel.
shape.front_linearity_r2	R^2^ of that leading-edge linear fit (planarity of the onset).
shape.front_curvature	Quadratic-term coefficient of a degree-2 fit to the leading edge.
shape.tail_slope_ms_per_ch	As front_slope, computed for the last-active-sample (trailing) edge.
shape.tail_linearity_r2	R^2^ of the trailing-edge linear fit.
shape.tail_curvature	Quadratic-term coefficient of the trailing-edge fit.
**Moveout (mov)—2 features**	
mov.xcorr_r2	R^2^ of a linear fit of cumulative inter-channel time lags (from FFT cross-correlation of adjacent active channels, maximum lag 30 samples, or a quarter of the trigger duration if shorter) versus channel position.
mov.xcorr_slope	Slope of that fit converted to apparent slowness (ms per metre); its sign gives the propagation direction.
**Background (bg)—3 features**	
bg.rms	RMS amplitude of the 30 s pre-trigger background window over the active channels.
bg.spectral_centroid_hz	Spectral centroid (power-weighted mean frequency) of the mean background trace.
bg.channel_cv	Coefficient of variation of the per-channel background RMS.
**Spectral (spec)—5 features**	
spec.valid	1 if the trigger is long enough to transform reliably, else 0 (the other spec features are then set to 0).
spec.centroid_hz	Spectral centroid of the mean active-channel trace within the ROI (single FFT).
spec.bandwidth_hz	Power-weighted standard deviation of frequency about the centroid.
spec.rolloff_85_hz	Frequency below which 85% of the cumulative power lies.
spec.flatness	Spectral flatness: geometric mean/arithmetic mean of the power spectrum (tonal vs. broadband).
**Coherence (coh)—3 features**	
coh.neighbor_corr_mean	Mean normalised cross-correlation of adjacent active-channel waveform pairs.
coh.neighbor_corr_median	Median of those adjacent-pair correlations.
coh.corr_decay_rate	Negative slope of mean correlation versus channel offset (offsets 1, 2, 4, 8): the rate at which coherence decays with separation.
**Fragmentation (frag)—2 features**	
frag.n_components	Number of connected components in the mask (4-connectivity).
frag.largest_component_fraction	Fraction of body_area in the largest connected component (1 = a single body).
**Quality (qual)—4 features**	
qual.mask_touches_t0	1 if the trigger touches the chunk start in time (possible truncation).
qual.mask_touches_t1	1 if the trigger touches the chunk end in time.
qual.mask_touches_ch0	1 if the trigger touches the first analysed channel.
qual.mask_touches_ch1	1 if the trigger touches the last analysed channel. These flags mark possibly truncated triggers and are excluded from the similarity and scaling subspaces.
**Context (ctx)—3 features**	
ctx.gap_before_ms	Time gap to the nearest preceding trigger of the same detector (capped at 60 s).
ctx.gap_after_ms	Time gap to the nearest following trigger of the same detector (capped at 60 s).
ctx.density_per_min	Number of same-detector triggers within ±120 s, expressed per minute.

## Appendix C. Feature Subspaces of the Machine-Learning Gates

This appendix lists the exact feature subspace used by each machine-learning gate in the final-pass compositions of this study (Section 2.3.3), as selected by the task-specific screening of Section 2.3.2. The 19 filter compositions evaluated during the campaign (Section 3.2) are candidate filter chains assembled from the three gate families. Table A2 lists the individual gate configurations of the final-pass composition of each stream, together with the seed-only illustrative gate shown in Figure 5. The catalogue accumulated over the whole campaign rather than from any single configuration, and the gate toolbox itself grew during the search as recurrent noise families were labelled (Section 2.3.4). The final-pass compositions therefore document each gate family in its most developed form. Earlier passes used feature subspaces selected by the same screening procedure and, in some cases, a different set of stages. On CRC4 STA/LTA, for example, intermediate passes included cluster and supervised gates, while the final pass ranked the stream by retrieval alone. Feature definitions are given in Table A1.

**Table A2:** Feature subspaces of the machine-learning gates used in this study (final-pass configurations; the gate shown in Figure 5 is a seed-only illustration). Feature names follow Table A1.

Gate (Configuration)	Stream	Feature Subspace
Unsupervised cluster gate (k-means, k = 6; KS-ranked top-10 subspace)	CRC4 semblance	mask.coincidence_efficiency, mask.triggered_channels, mask.back_loaded_ratio, mask.coincidence_mean, geo.channel_span_m, env.temporal_entropy, shape.tail_curvature, mask.coincidence_peak, mask.temporal_consistency, mask.fill_ratio
Unsupervised cluster gate (k-means, k = 6; score-ranked top-16 subspace)	CRC7 semblance	mask.triggered_channels, geo.center_depth_m, ctx.density_per_min, mask.coincidence_mean, wave.roi_peak_abs, wave.roi_crest_factor, mask.coincidence_efficiency, mask.body_area, ctx.gap_before_ms, shape.front_curvature, shape.tail_curvature, ctx.gap_after_ms, mask.max_channel_gap_m, env.rise_time_ms, shape.tail_slope_ms_per_ch, shape.tail_linearity_r2
Cluster gate shown in Figure 5 (seed-only illustration; k-means, k = 6; score-ranked top-4 subspace)	CRC4 semblance	mask.triggered_channels, mask.coincidence_efficiency, mask.back_loaded_ratio, env.temporal_entropy
Retrieval ranking (Manhattan distance; 3-nearest-neighbour mean prototype)	CRC7 semblance	geo.center_depth_m, mask.triggered_channels, ctx.density_per_min
Retrieval ranking (Manhattan distance; 3-nearest-neighbour mean prototype; final pass applied without a preceding cluster gate)	CRC4 STA/LTA	geo.center_depth_m, mask.temporal_consistency, wave.channel_rms_cv, mask.spatial_continuity, shape.front_linearity_r2, mov.xcorr_r2, mask.coincidence_mean, bg.channel_cv, shape.tail_linearity_r2, coh.neighbor_corr_mean, mask.back_loaded_ratio, ctx.density_per_min, spec.bandwidth_hz, ctx.gap_before_ms, coh.corr_decay_rate, amp.crest_factor, frag.largest_component_fraction, amp.snr_rms, wave.roi_skewness, mov.xcorr_slope, shape.front_curvature
Supervised noise gate: orbital-pattern (noise-centroid distance rule)	CRC4 semblance	wave.channel_rms_cv, geo.aspect_ratio, shape.front_linearity_r2, spec.flatness, bg.spectral_centroid_hz, mask.coincidence_mean, mask.temporal_uniformity, geo.channel_span_m, amp.crest_factor, mask.back_loaded_ratio, ctx.gap_after_ms, frag.largest_component_fraction, spec.rolloff_85_hz, spec.bandwidth_hz, mask.spatial_continuity, spec.centroid_hz, mask.solidity, shape.front_curvature, mov.xcorr_slope, mask.front_loaded_ratio, mov.xcorr_r2, wave.roi_rms, env.peak_time_ratio, ctx.gap_before_ms
Supervised noise gate: common-mode array (noise-centroid distance rule)	CRC4 semblance	env.energy_half_width_ms, wave.time_rms_cv, frag.largest_component_fraction, mask.temporal_uniformity, mask.coincidence_efficiency, amp.channel_energy_cv, shape.front_linearity_r2, mask.spatial_continuity, geo.center_depth_m, amp.snr_rms, mask.solidity, mask.body_area, coh.corr_decay_rate, bg.rms, mask.triggered_channels
Supervised noise gate: surface (noise-centroid distance rule)	CRC4 semblance	amp.channel_energy_cv, mask.coincidence_efficiency, ctx.gap_before_ms, mask.back_loaded_ratio, frag.largest_component_fraction
Supervised noise gate: general (noise-centroid distance rule)	CRC4 semblance	ctx.gap_before_ms, amp.snr_rms, shape.front_linearity_r2, frag.largest_component_fraction, amp.rms_signal, mask.back_loaded_ratio
Gradient-boosted binary classifier (LightGBM; rejection threshold set on held-out labels)	CRC4 semblance	mask.coincidence_efficiency, frag.largest_component_fraction, amp.channel_energy_cv, ctx.gap_before_ms, env.temporal_entropy, geo.center_depth_m, mask.coincidence_mean, mask.triggered_channels, frag.n_components, amp.snr_rms, geo.aspect_ratio, mask.back_loaded_ratio, shape.tail_curvature, mask.spatial_continuity, mask.solidity, shape.front_curvature, shape.front_linearity_r2, ctx.gap_after_ms, ctx.density_per_min, shape.front_slope_ms_per_ch, mov.xcorr_slope, coh.corr_decay_rate, mask.temporal_consistency, env.energy_half_width_ms, shape.tail_slope_ms_per_ch, geo.duration_ms, shape.tail_linearity_r2, wave.roi_skewness
